# The Preparation of Glabridin-Loaded Liposomes and Their Inhibition Effects on Melanogenesis

**DOI:** 10.3390/molecules31183179

**Published:** 2026-09-10

**Authors:** Ling Zeng, Peng Gao, Jing Zhou, Ping Zhang, Ding Ma, Jinfang Zhu

**Affiliations:** 1 College of Food Science and Pharmacy, Xinjiang Agricultural University, Urumqi 830052, China; 15310495394@163.com (L.Z.); gaopeng9144911@sina.com (P.G.); zp97062022@126.com (P.Z.); gertaya@163.com (D.M.); 2 Xinjiang Uygur Autonomous Region Special Fruits and Vegetables Processing Engineering Research Center, Urumqi 830052, China; 3 Xinjiang Longhuiyuan Pharmaceutical Co., Ltd., Tacheng 834700, China; 17347761385@163.com

**Keywords:** Glabridin, liposomes, stability, anti-melanogenesis

## Abstract

Glabridin (GLA) is widely applied in cosmetics, pharmaceuticals, and food products; however, its application is limited by poor aqueous solubility, low stability, and low bioavailability. To overcome these disadvantages, Glabridin-Loaded Liposomes (L-GLAs) were prepared using a thin-film hydration–solvent evaporation method combined with high-pressure homogenization. The cumulative release rate of L-GLA within 96 h was higher than that of GLA. The stability of L-GLA was superior to that of GLA. The inhibitory effects and underlying mechanisms of GLA and L-GLA on B16 mouse melanoma cells were investigated. The IC_50_ of L-GLA against B16 melanoma cells (84.66 μM) was lower than that of GLA (115.5 μM). Both compounds induced apoptosis in B16 cells, with L-GLA exhibiting greater potency. This effect may be related to the inhibition of B16 cell proliferation via G0/G1 phase arrest. Furthermore, in a melanin-producing B16 cell model, the melanin content and tyrosinase (TYR) activity in the GLA and L-GLA groups were lower than those in the model group, and L-GLA exerted a stronger inhibitory effect than GLA. Quantitative PCR analysis revealed that GLA and L-GLA reduced the mRNA expression levels of MITF, TYR, TRP-1, and TRP-2; and Western blotting further confirmed that the protein levels of these melanogenesis-related factors were consistently downregulated in B16 cells following photodynamic treatment. Mechanistically, these anti-melanogenic effects appeared to be associated with the downregulation of MITF and its downstream targets, potentially through the PKA/MITF and MAPK/MITF signaling cascades. Collectively, these findings suggest that liposomal encapsulation may enhance the anti-melanogenic and anti-proliferative bioactivity of GLA, which implies its potential as an improved formulation for biomedical and cosmetic applications.

## 1. Introduction

Glabridin (GLA) is a typical flavonoid compound with the molecular formula C_20_H_20_O_4_ and a molecular weight of 324.37 g/mol, and is predominantly found in *Glycyrrhiza glabra* [1,2,3]. The content of GLA in the *Glycyrrhiza glabra* is approximately 0.1–0.3% [4]. GLA exhibits anti-melanogenic, anti-oxidative and anti-inflammatory activities and is widely used in cosmetics, functional foods, and medicines [5,6]. Specifically, the anti-melanogenic activity is primarily mediated through the direct suppression of tyrosinase (TYR) activity and the downregulation of melanogenic gene expression, which are considered important for inhibiting hyperpigmentation disorders [7]. Its antioxidant capacity arises from the ability to scavenge free radicals and inhibit lipid peroxidation, thereby providing robust cellular protection against oxidative stress [8]. Additionally, its anti-inflammatory effects are exerted by suppressing the production of pro-inflammatory cytokines, including tumor necrosis factor-alpha (TNF-α) and interleukin-6 (IL-6), and by modulating the nuclear factor-kappa B (NF-κB) signaling pathway [9]. Despite these bioactivities, the practical application of GLA is severely constrained by its poor aqueous solubility, chemical instability, and low oral bioavailability [4,10]. To surmount these physicochemical and biopharmaceutical limitations, liposomal encapsulation has emerged as a promising strategy. Liposomes are spherical vesicles composed of self-assembled phospholipid bilayers, in which hydrophilic phosphate head groups face the aqueous environment while hydrophobic acyl chains constitute the membrane interior [11,12,13]. This amphiphilic architecture enables the encapsulation of hydrophobic bioactive compounds within the lipid bilayer or hydrophobic core, thereby enhancing their physicochemical stability and bioaccessibility [14,15]. Consequently, Glabridin-Loaded Liposomes (L-GLAs) have been increasingly investigated as delivery systems in functional foods, pharmaceuticals, and cosmetics, owing to their favorable biocompatibility, biodegradability, and targeted delivery potential [15,16,17]. These attributes suggest that L-GLA holds promise in several fields: in cosmetic formulations, L-GLA has been evaluated for its capacity to enhance transdermal penetration and improve depigmenting efficacy [18]; in pharmaceutical research, L-GLA has been explored for its potential to increase bioavailability and facilitate targeted delivery in anti-inflammatory and antitumor applications [19]. In the context of functional foods, liposomal encapsulation has been reported to protect GLA against degradation and to facilitate its intestinal absorption [20]. Collectively, these findings indicate that liposomal encapsulation represents a rational strategy to circumvent the inherent physicochemical limitations of GLA and potentiate its bioactivity. In the present study, L-GLA was prepared using a combination of the film hydration method and high-pressure homogenization. This preparation strategy differs from most previously reported protocols, which typically rely on film hydration alone and yield liposomes with average particle sizes of approximately 200 nm [21]. By incorporating high-pressure homogenization, the high shear forces generated during the process effectively break down multilamellar vesicles into smaller and more uniform particles. Given that anti-melanogenesis represents one of the most prominent bioactivities of GLA, these improved physicochemical properties are expected to contribute to enhanced anti-melanogenic efficacy. However, the molecular targets and signaling pathways underlying this enhanced activity remain to be fully elucidated.

An understanding of melanin synthesis regulation is important for elucidating how GLA and L-GLA exert their anti-melanogenic effects. Mechanistically, the anti-melanogenic activity of GLA is closely associated with the regulation of melanin synthesis pathways. Melanin synthesis is a complex biological process involving the synergistic action of multiple factors, including tyrosinase (TYR), and its tyrosinase-related proteins TRP-1 and TRP-2 [22,23]. TYR is a key enzyme in melanin synthesis and promotes melanin production [24]. The expression of TYR is regulated by the microphthalmia-associated transcription factor (MITF) [25,26]. MITF, as a melanocyte-specific transcription factor, directly activates the promoter of TYR, and upregulates the expression of TRP-1 and TRP-2, thereby promoting melanin production [27,28,29]. Previous studies have demonstrated that GLA can inhibit melanin synthesis by suppressing TYR activity [3]. According to the literature [30], proanthocyanidins inhibited melanin production via the suppression of TYR and TRP-1. Shu et al. [31] found that Arbutin inhibited TYR activity, thereby preventing melanin production and reducing skin pigmentation. Nevertheless, the specific effects of GLA, and particularly those of its liposomal formulation, on MITF-driven transcriptional regulation and the upstream signaling cascades involved remain insufficiently characterized.

In this study, GLA was encapsulated into L-GLA to overcome these limitations, including poor aqueous solubility, instability, and low bioavailability. Specifically, we investigated the in vitro release behavior, stability under various temperature conditions, and degradation kinetics of both formulations. Moreover, we examined their effects on B16 mouse melanoma cells, including cytotoxicity, apoptosis, and cell cycle distribution, with a particular emphasis on melanin content, TYR activity, and the mRNA and protein expression of MITF and its downstream targets: TYR, TRP-1, and TRP-2. This assessment aims to provide preliminary insights into the enhanced anti-melanogenic efficacy of liposomal GLA and to support its further development as a functional ingredient in biomedical and cosmetic applications.

## 2. Results

### 2.1. Preparation and Characterization of L-GLA

**Figure 1:**
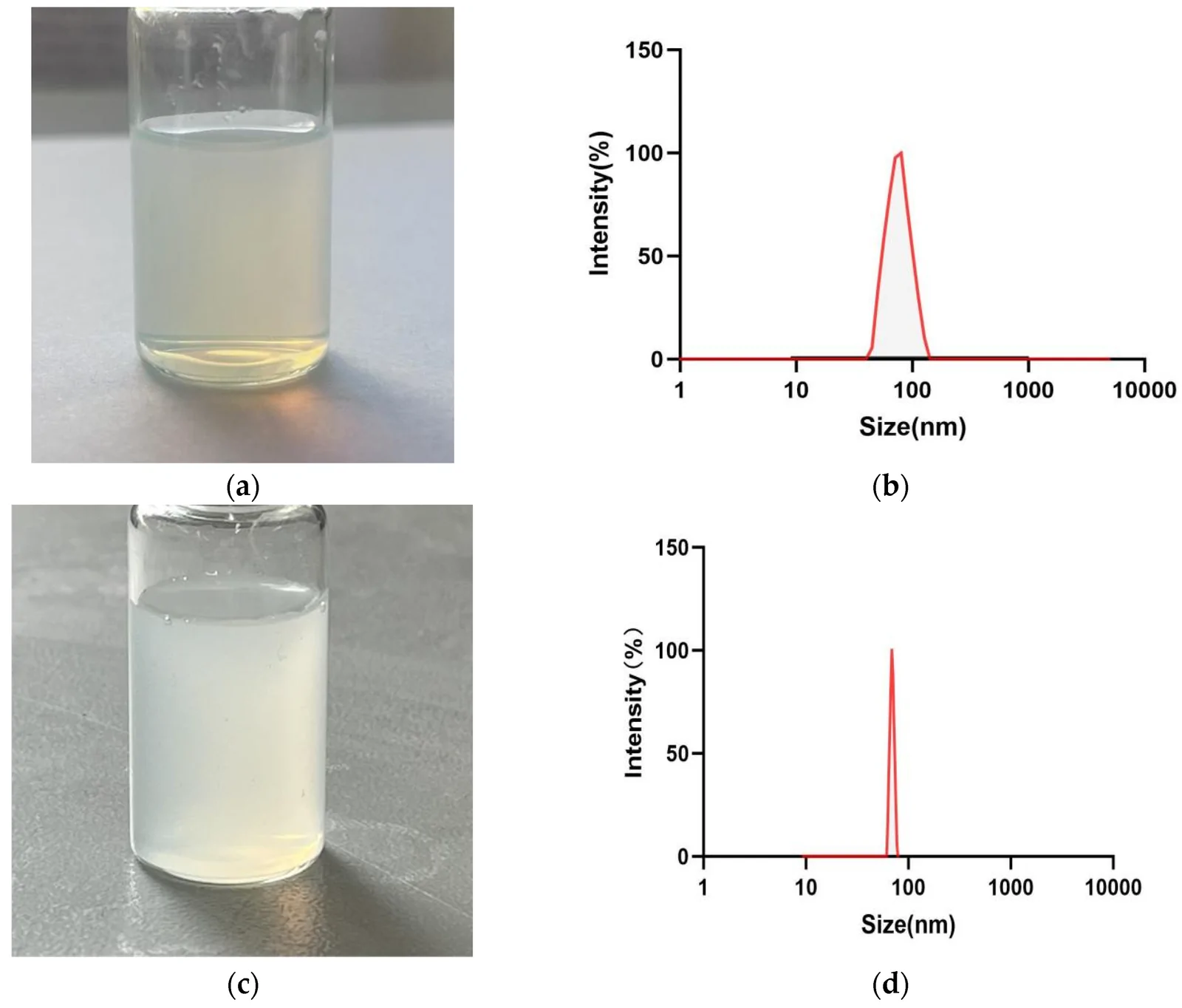
Characterization of L-BLANK and L-GLA. (**a**) Photograph of L-BLANK solution. (**b**) Particle size distribution of L-BLANK determined by dynamic light scattering (DLS). (**c**) Photograph of L-GLA solution. (**d**) Particle size distribution of L-GLA determined by DLS.

**Figure 2:**
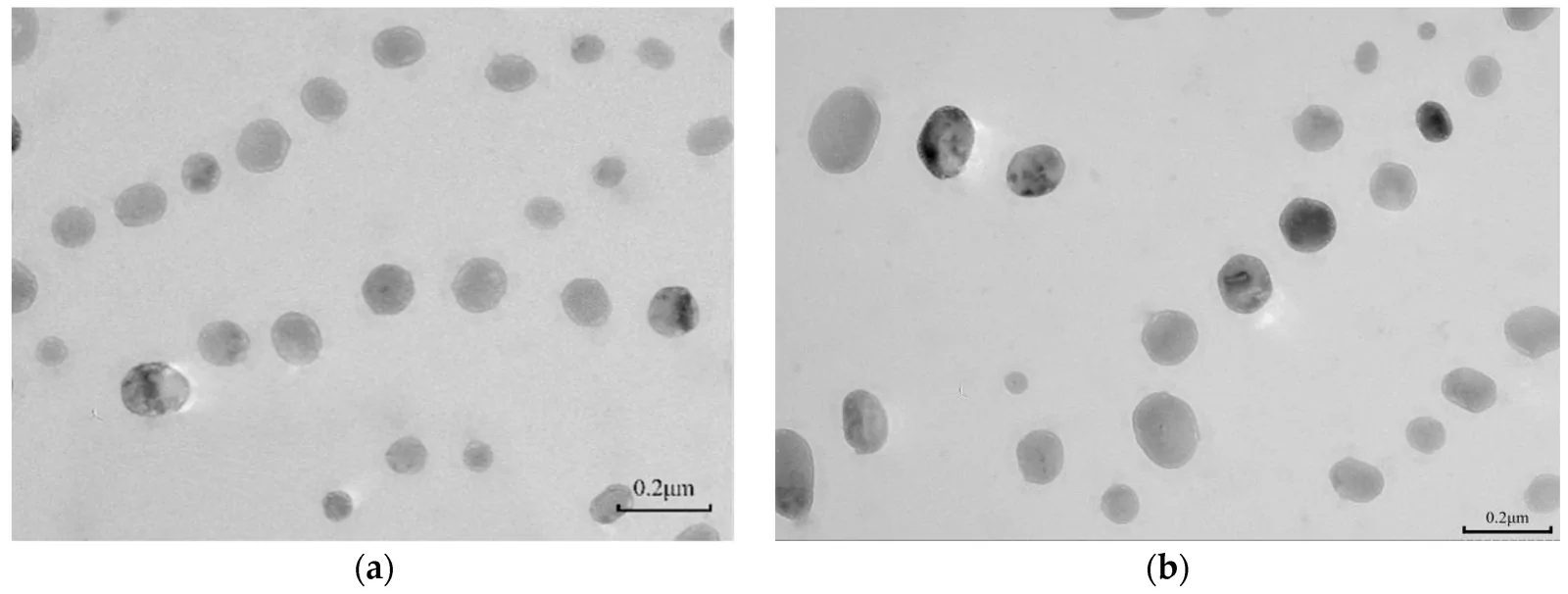
TEM analysis of L-BLANK (**a**) and L-GLA (**b**); scale bar = 0.2 μm.

The particle size of L-GLA exhibited a relatively homogeneous distribution with an average diameter of 50.24 ± 1.15 nm, a PDI of 0.297 ± 0.01, and a Zeta potential of −3.44 ± 0.04 mV (Figure 1). Similarly, L-BLANK showed a homogeneous distribution, with an average diameter of 44.60 ± 0.68 nm, a PDI of 0.296 ± 0.01, and a Zeta potential of −2.24 ± 0.40 mV (Figure 1). TEM analysis further revealed that L-GLA and L-BLANK possessed a homogeneous spherical morphology and uniform particle sizes (Figure 2). The loading capacity (LC) and encapsulation efficiency (EE) of L-GLA were 4.38% and 80.71%, respectively. Thus, GLA was encapsulated in the hydrophobic membrane of liposomes.

### 2.2. The Release Profiles of GLA and L-GLA In Vitro

**Figure 3:**
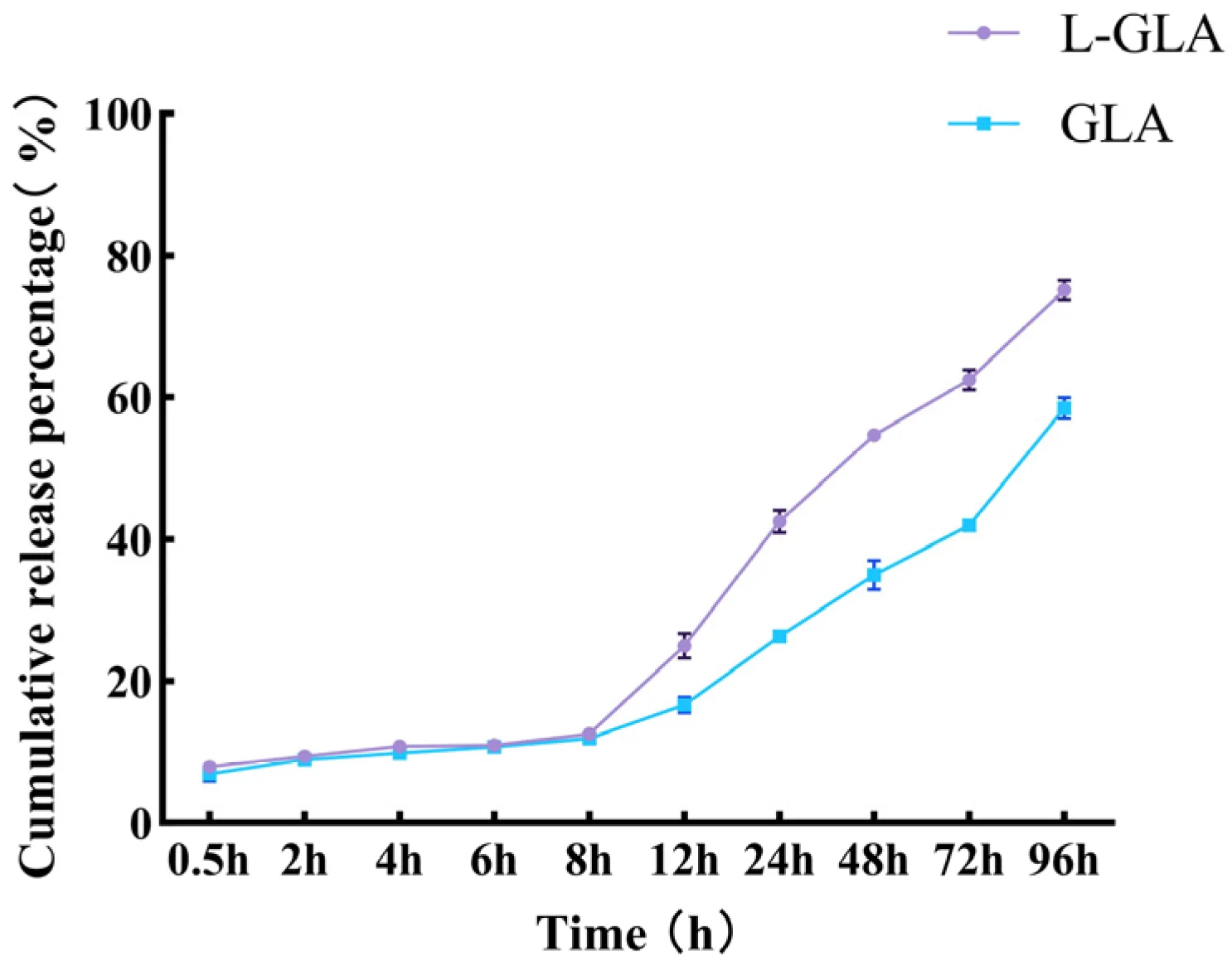
The release profiles of GLA and L-GLA.

As shown in Figure 3, the cumulative release percentages of L-GLA (75.11%) were higher than those of GLA (58.48%) within 96 h, suggesting that the liposomal formulation may enhance the in vitro release rate of free GLA. The release percentages of L-GLA did not exceed 40% within 0.5 h, suggesting the absence of a substantial burst release phenomenon. Given that release behavior is related to bioavailability, this observation suggests that L-GLA may have the potential to improve the bioavailability of GLA.

**Figure 4:**
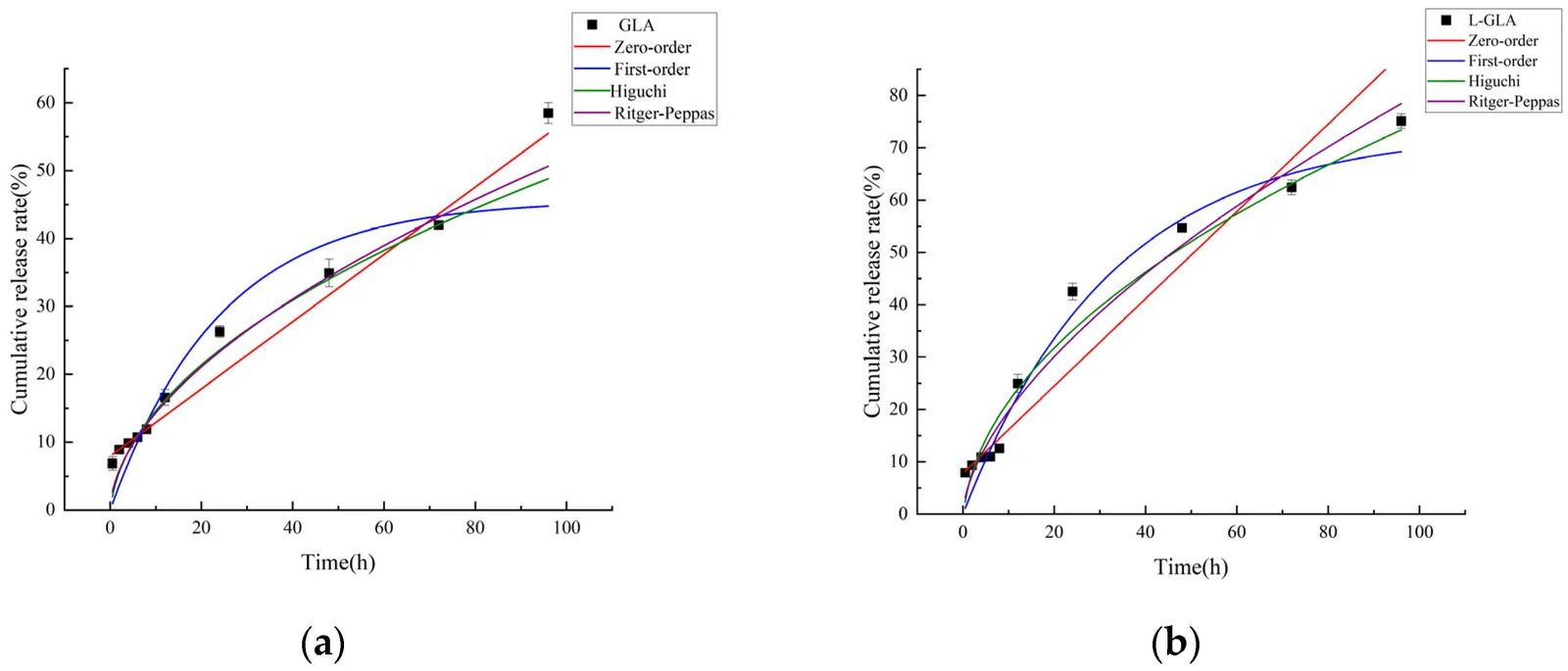
In vitro release curves of GLA (**a**) and L-GLA (**b**) fitted with various kinetic models.

To comprehensively investigate the release behaviors of free GLA and L-GLA, four commonly employed kinetic models—zero-order, first-order, Higuchi, and Ritger–Peppas—were applied to fit the cumulative release data. The results are presented in Figure 4 and Table 1. Comparison of the coefficients of determination (R^2^) for the four models showed that, for the release of free GLA from the GLA formulation, the highest R^2^ value (0.9785) was obtained with the zero-order kinetic model. In contrast, as shown in Table 2, the highest R^2^ value (0.9556) for the release of GLA from L-GLA was obtained with the Ritger–Peppas kinetic model. These findings suggest that the cumulative release of free GLA from the GLA formulation follows zero-order kinetics, with the release process occurring primarily at a constant rate [32]. Conversely, the release of GLA from L-GLA conforms to the Ritger–Peppas kinetic model, suggesting that GLA may be uniformly loaded into the phospholipid bilayer of the liposomes and that the release mechanism involves a combination of dissolution and diffusion [33].

### 2.3. Establishment of a Temperature Degradation Kinetic Model

**Figure 5:**
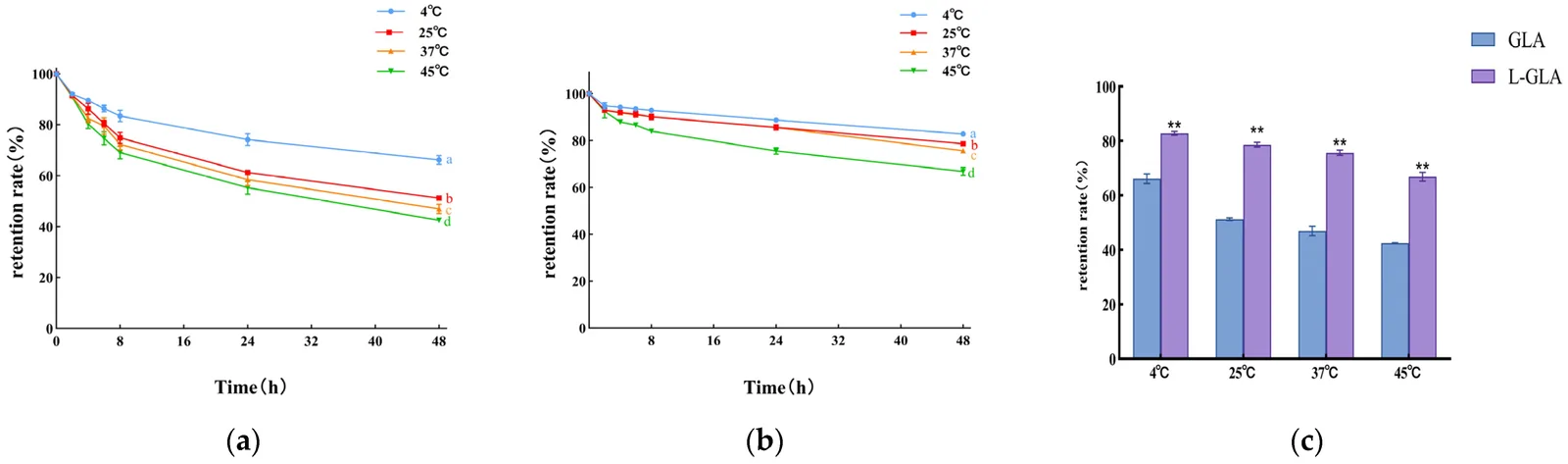
Temperature-dependent retention of GLA in GLA (**a**) and L-GLA (**b**) and their comparison (**c**). Annotations: Different lowercase letters (a–d) indicate significant differences among groups at 48 h. Compared with the GLA group; ** *p* < 0.01.

As shown in Figure 5a,b, after GLA and L-GLA were stored for 48 h at different temperatures, their retention rates followed the same descending order: 4 °C (66.13% and 82.81%, respectively) > 25 °C (51.23% and 79.56%) > 37 °C (46.96% and 78.61%) > 45 °C (42.54% and 59.22%). These results suggest that, for both GLA and L-GLA, the retention rates decreased with increasing storage temperature. As shown in Figure 5c, the retention rate of L-GLA was higher than that of the GLA raw material at all storage temperatures (*p* < 0.01). This suggests that encapsulating GLA into liposomes may contribute to its enhanced stability.

### 2.4. Establishment of a Temperature Degradation Kinetic Model

**Figure 6:**
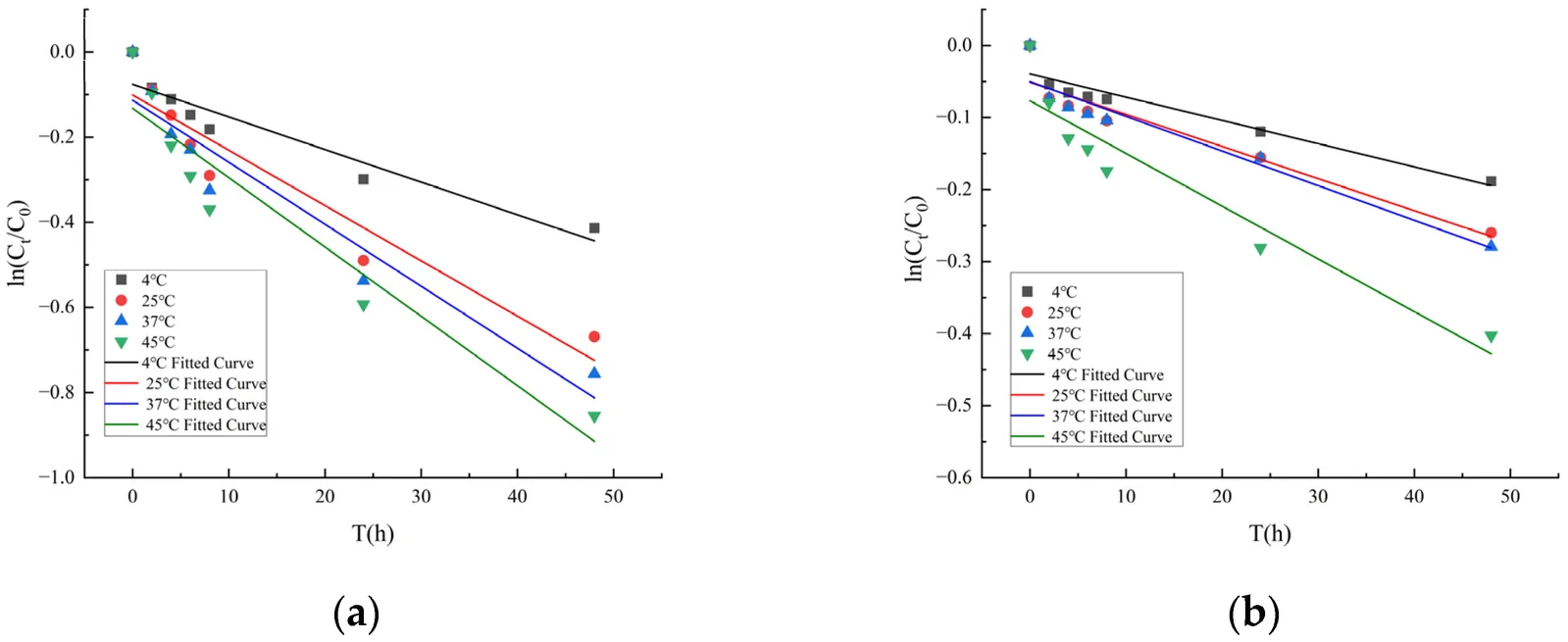
Temperature degradation kinetic curves of GLA in GLA (**a**) and L-GLA (**b**).

To obtain the degradation kinetics curves for GLA and L-GLA, time (t) was plotted on the x-axis against the natural logarithm of the concentration ratio, ln(C_t_/C_0_), on the y-axis (Figure 6). Each curve was fitted to determine the correlation coefficient (R^2^). The degradation rate constant (k) was subsequently calculated using the first-order reaction kinetics equation. By plotting the natural logarithm of the degradation rate constant (ln k) against the reciprocal of absolute temperature (1/T), the apparent activation energy (Ea) for both GLA and L-GLA was derived from the slope of the resulting linear plot. The correlation coefficient (R^2^) serves as a metric for evaluating the quality of curve fitting, with values approaching unity (1) indicating a better fit. The degradation rate constant (k) provides a quantitative measure of the reaction rate, where a larger k value corresponds to a faster degradation process [34]. The apparent activation energy (Ea) reflects the kinetic barrier of the degradation reaction; a higher Ea value suggests that the reaction proceeds less readily [35]. As presented in Table 3, the R^2^ values for the degradation kinetics fitting curves of both GLA and L-GLA exceeded 0.9, suggesting that their thermal degradation processes followed first-order reaction kinetics. At different temperatures, the k values (h^−1^) for GLA and L-GLA ranked as follows: 4 °C (0.008 and 0.003, respectively) < 25 °C (0.013 and 0.004) < 37 °C (0.015 and 0.005) < 45 °C (0.016 and 0.007). These results suggested that higher temperatures were associated with larger k values, consistent with the observation that GLA degraded more rapidly at elevated temperatures. At all temperatures tested, the k values for L-GLA were lower than those for GLA, suggesting that encapsulation of GLA into liposomes enhances its stability under the conditions tested. Furthermore, the higher Ea value determined for L-GLA, relative to that of GLA, suggests a higher energy barrier to degradation, which may render the liposomal formulation kinetically more stable under the tested conditions.

Enthalpy change (ΔH) is a thermodynamic quantity that describes the heat absorbed or released during a reaction at constant pressure [36]. Under specific conditions, the endothermic or exothermic nature of a reaction depends on the enthalpy difference between products and reactants (i.e., ΔH). Positive ΔH values correspond to endothermic reactions; negative values, to exothermic reactions. As shown in Table 4, the ΔH values for both GLA and L-GLA were positive, suggesting that the degradation of GLA in both forms is an endothermic reaction. The ΔH values for L-GLA were higher than those for GLA, suggesting that the system requires a greater amount of energy during the degradation process. This finding raises the possibility that L-GLA possesses improved stability relative to GLA under the present experimental conditions.

The Gibbs free energy change (ΔG) represents the difference in free energy between the products and the reactants [37]. A positive ΔG indicates that the degradation process is non-spontaneous. Since the ΔG values for both GLA and L-GLA were positive, both degradation processes are considered non-spontaneous.

### 2.5. Cytotoxicity Test

**Figure 7:**
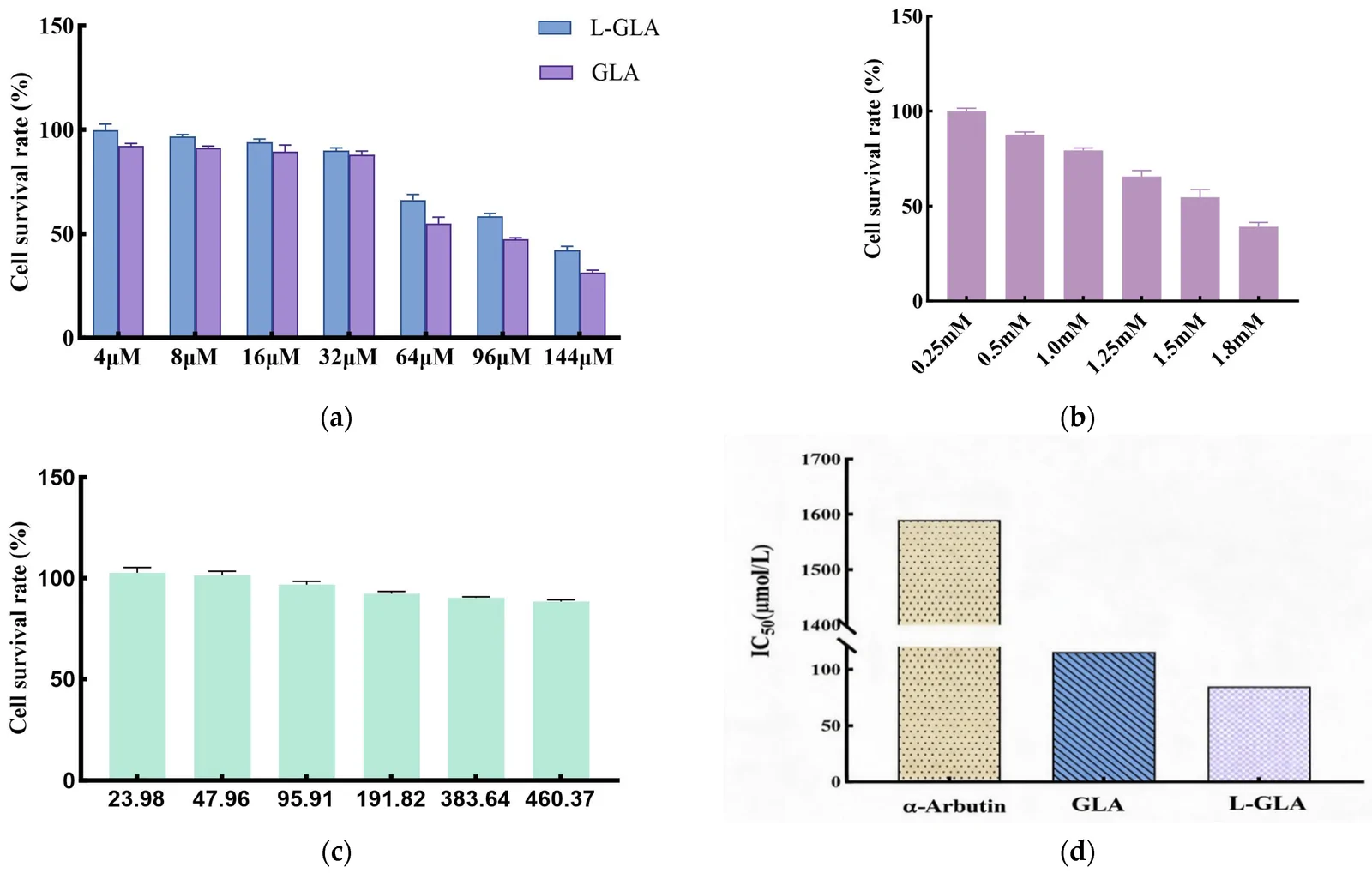
Cytotoxic effects of GLA, L-GLA, α-arbutin, and L-BLANK on B16 cells. (**a**) Dose–response curves of GLA and L-GLA; (**b**) dose–response curve of α-arbutin; (**c**) dose–response curve of L-BLANK; (**d**) IC_50_ values of GLA, L-GLA, and α-arbutin. Cell viability is expressed as a percentage of the control level.

The cytotoxicity of GLA, L-GLA, α-arbutin, and L-BLANK against B16 cells was evaluated using the MTT assay. As shown in Figure 7, at the same concentration of GLA, the cell survival rate in the L-GLA group was lower than that in the GLA group. Furthermore, the IC_50_ value of L-GLA against B16 cells (84.66 μM) was lower than that of GLA (115.5 μM). Additionally, the IC_50_ value of α-arbutin (1590 μM) was higher than that of GLA and L-GLA, indicating that the cytotoxicity of GLA and L-GLA was greater than that of α-arbutin. Moreover, at concentrations not exceeding 32 μM for GLA and L-GLA, and 0.5 mmol/L for α-arbutin, the cell survival rate exceeded 85%. Therefore, GLA at 32 μM and α-arbutin at 0.5 mmol/L were used in subsequent cellular experiments to investigate the mechanism of melanin inhibition, ensuring a sufficient number of viable cells. When the concentration of the carrier material (phospholipids and cholesterol at a 30:1 ratio) in L-BLANK reached 460.37 μg/mL, the viability of B16 cells exceeded 85%. The liposomal carrier exhibited low cytotoxicity and did not significantly interfere with the cytotoxicity of L-GLA or the subsequent cellular assays.

### 2.6. Flow Cytometric Analysis of Cell Cycle Distribution and Apoptosis

#### 2.6.1. Cell Cycle Analysis

**Figure 8:**
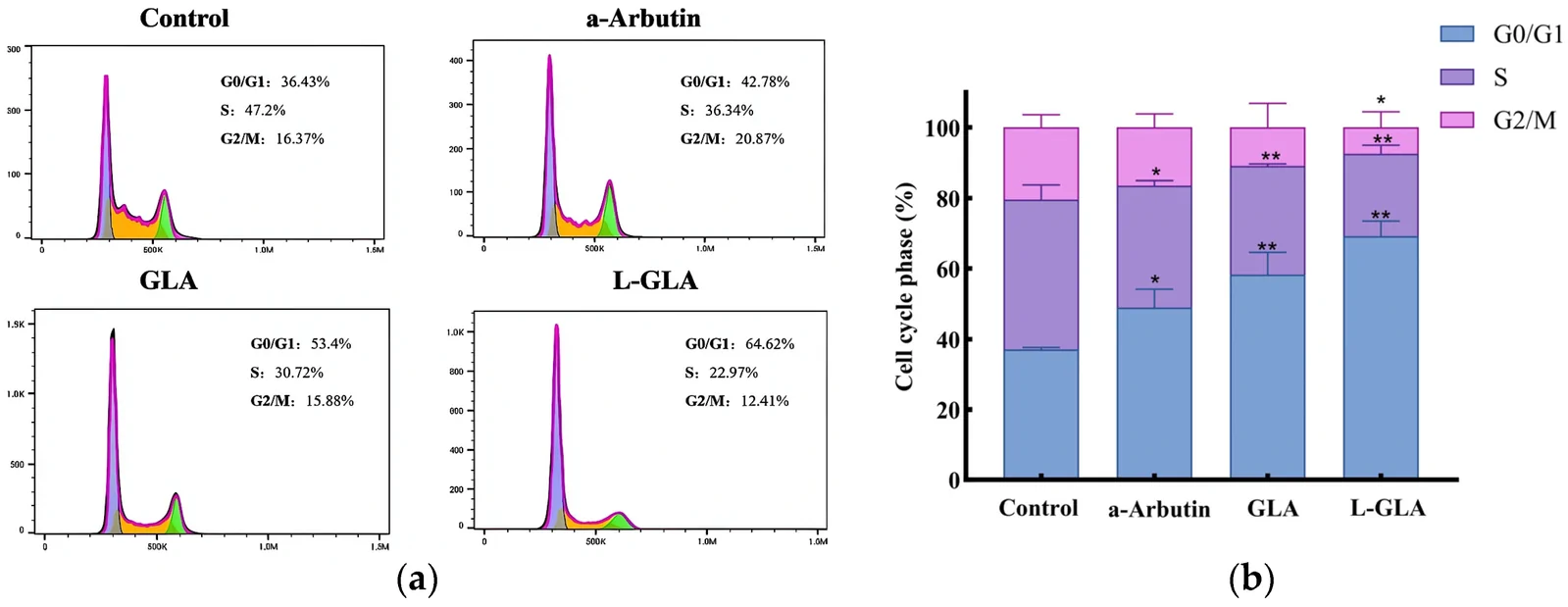
Effects of GLA, L-GLA, and α-arbutin on cell cycle distribution in B16 cells. (**a**) Representative flow cytometry histograms of cell cycle phase distribution after treatment with GLA, L-GLA, and α-arbutin; (**b**) quantitative analysis of the percentage of cells in G0/G1, S, and G2/M phases. Compared with the Control group, * *p* < 0.05, ** *p* < 0.01.

The cell cycle includes the first gap phase (G1), the DNA synthesis phase (S), the second gap phase (G2), the mitotic phase (M), and the quiescent phase (G0) [38]. The percentage of cells in G0/G1 phase reflects the proportion of cells in either the G0 or G1 phase and serves as an indicator of cellular proliferation activity, with a higher percentage indicating lower proliferative activity. The percentage of cells in S phase reflects the proportion of cells undergoing DNA synthesis and serves as an indicator of DNA replication activity: the higher the percentage, the higher the replication activity. The percentage of cells in G2/M phase reflects the proportion of cells in either the G2 or M phase and serves as an indicator of cell division activity: the higher the percentage, the higher the division activity. A reduction in the percentage of cells in S phase or G2/M phase suggests that a treatment may exert its effects by inhibiting proliferation or inducing apoptosis [39]. As shown in Figure 8, the percentages of cells in the G0/G1 phase were higher in the GLA, L-GLA, and α-arbutin groups than in the control group (*p* < 0.01 or *p* < 0.05), suggesting induction of G0/G1 phase arrest by these treatments. Conversely, the percentages of cells in the S phase were lower in all treatment groups compared with the control (*p* < 0.01 or *p* < 0.05), which may reflect an inhibitory effect on cell proliferation. Additionally, a lower percentage of cells in the G2/M phase was observed for the L-GLA group relative to the control (*p* < 0.05), potentially indicating an additional interference with cell division. Collectively, these findings indicate that GLA, L-GLA, and α-arbutin promote G0/G1 arrest to varying extents, accompanied by a reduction in S-phase cell populations, while L-GLA also appears to affect G2/M progression.

#### 2.6.2. Analysis of Apoptosis

**Figure 9:**
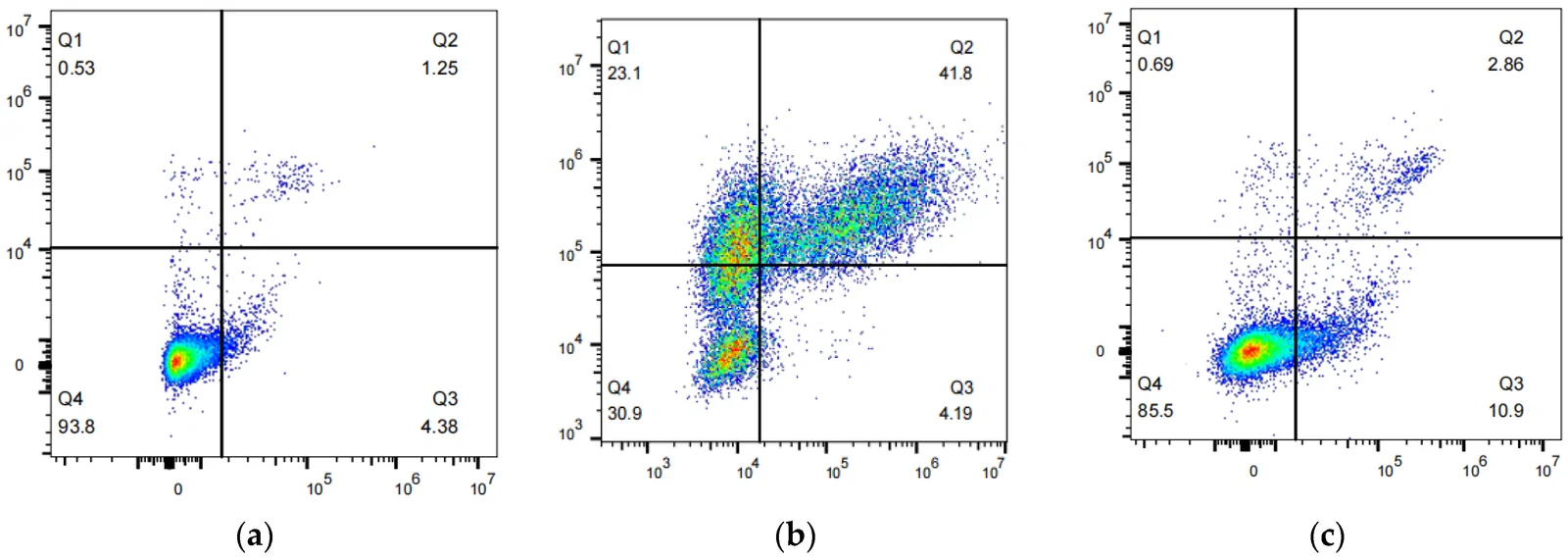

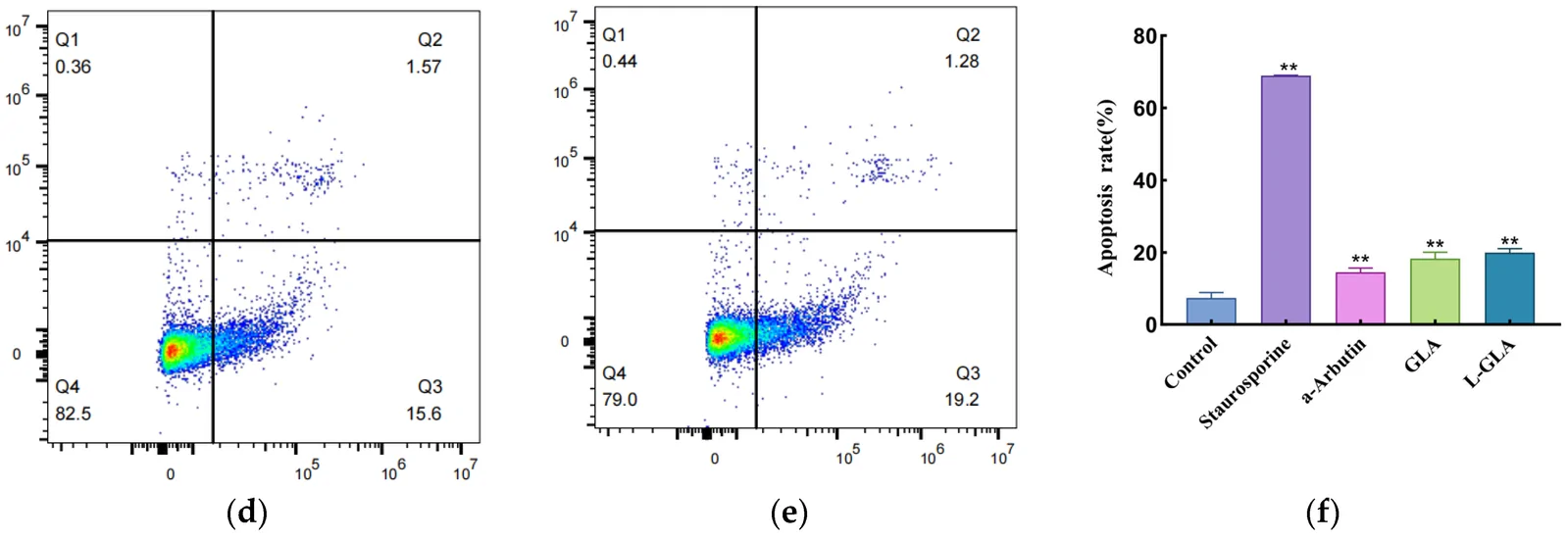
Effects of GLA, L-GLA, and α-arbutin on apoptosis in B16 cells. Staurosporine was used as a positive control. Apoptosis was detected by Annexin V-FITC/PI staining and flow cytometry. (**a**–**e**) Representative flow cytometric dot plots for the control, staurosporine, α-arbutin, GLA, and L-GLA groups, respectively. (**f**) quantification of apoptotic cell percentages (mean ± SD, n = 3). Compared with the Control group, ** *p* < 0.01.

To explore the effects of GLA and L-GLA on apoptosis, cells under different treatments were evaluated. The results are presented in the flow cytometry scatter plots (Figure 9a–e), where Q1 represents necrotic cells, Q2 represents late-stage apoptotic or necrotic cells, Q3 represents early-stage apoptotic cells, and Q4 represents normal viable cells [21]. Compared with the control group, the positive control group (staurosporine, 1 μM, 6 h) exhibited a marked increase in apoptotic cells, with a total apoptosis rate of 68.99% (*p* < 0.001), confirming the responsiveness and validity of the apoptosis assay system. The α-arbutin (0.5 mmol/L), GLA (32 μM), and L-GLA (32 μM) groups all showed increased proportions of early and late apoptotic cells, and as shown in Figure 9f, their total apoptosis rates were higher than that of the control group (*p* < 0.01), indicating that these treatments induce apoptosis in B16 cells under the experimental conditions tested.

### 2.7. Determination of TYR Inhibitory Activity of GLA and L-GLA In Vitro

**Figure 10:**
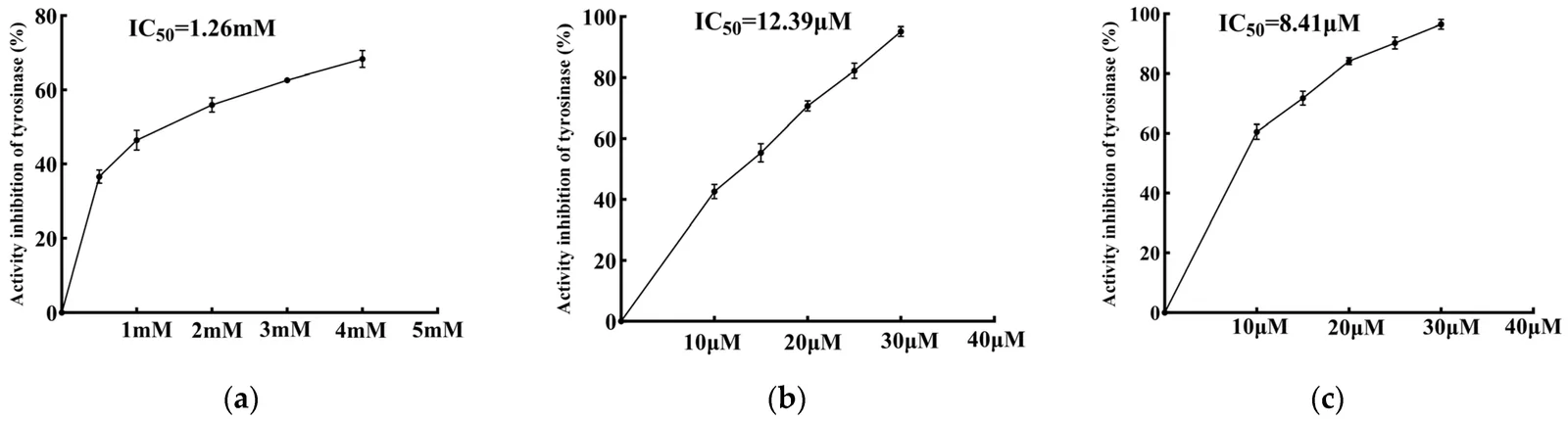

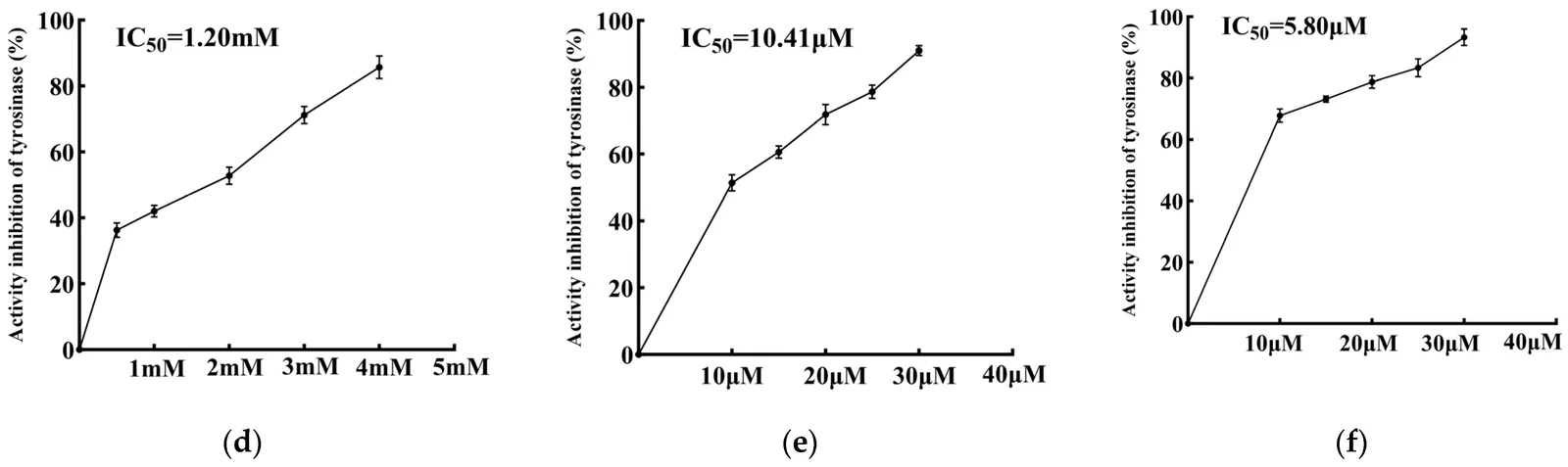
Dose-dependent inhibition of tyrosinase activity by α-arbutin, GLA, and L-GLA (from left to right). (**a**–**c**) Monophenolase activity inhibition curves with L-tyrosine as the substrate. (**d**–**f**) Diphenolase activity inhibition curves with L-DOPA as the substrate. The half-maximal inhibitory concentration (IC_50_) values were derived from the corresponding dose–response curves and are indicated in each panel. Data are presented as the mean ± standard deviation (SD).

The inhibitory effects of GLA and L-GLA against mushroom TYR were evaluated using both L-tyrosine (monophenolase) and L-DOPA (diphenolase) assays, with α-arbutin as the positive control (Figure 10). In the monophenolase assay, α-arbutin inhibited TYR activity by 68.32% at 4 mM, with an IC_50_ value of 1.26 mM. Under the same assay conditions, both GLA and L-GLA exhibited dose-dependent inhibition. At their respective maximum tested concentrations (30 μM), GLA and L-GLA achieved inhibition rates of 95.24% and 96.53%, with IC50 values of 12.39 μM and 8.41 μM, respectively (mean ± SD, n = 3). In the diphenolase assay, α-arbutin displayed an 85.74% inhibition rate and an IC_50_ of 1.20 mM, whereas GLA and L-GLA showed IC_50_ values of 10.41 μM and 5.80 μM, corresponding to inhibition rates of 91.08% and 93.37%, respectively. Collectively, these results indicate that both GLA and L-GLA are more potent TYR inhibitors than α-arbutin under the in vitro conditions tested.

### 2.8. Effects on Intracellular Melanin Production

**Figure 11:**
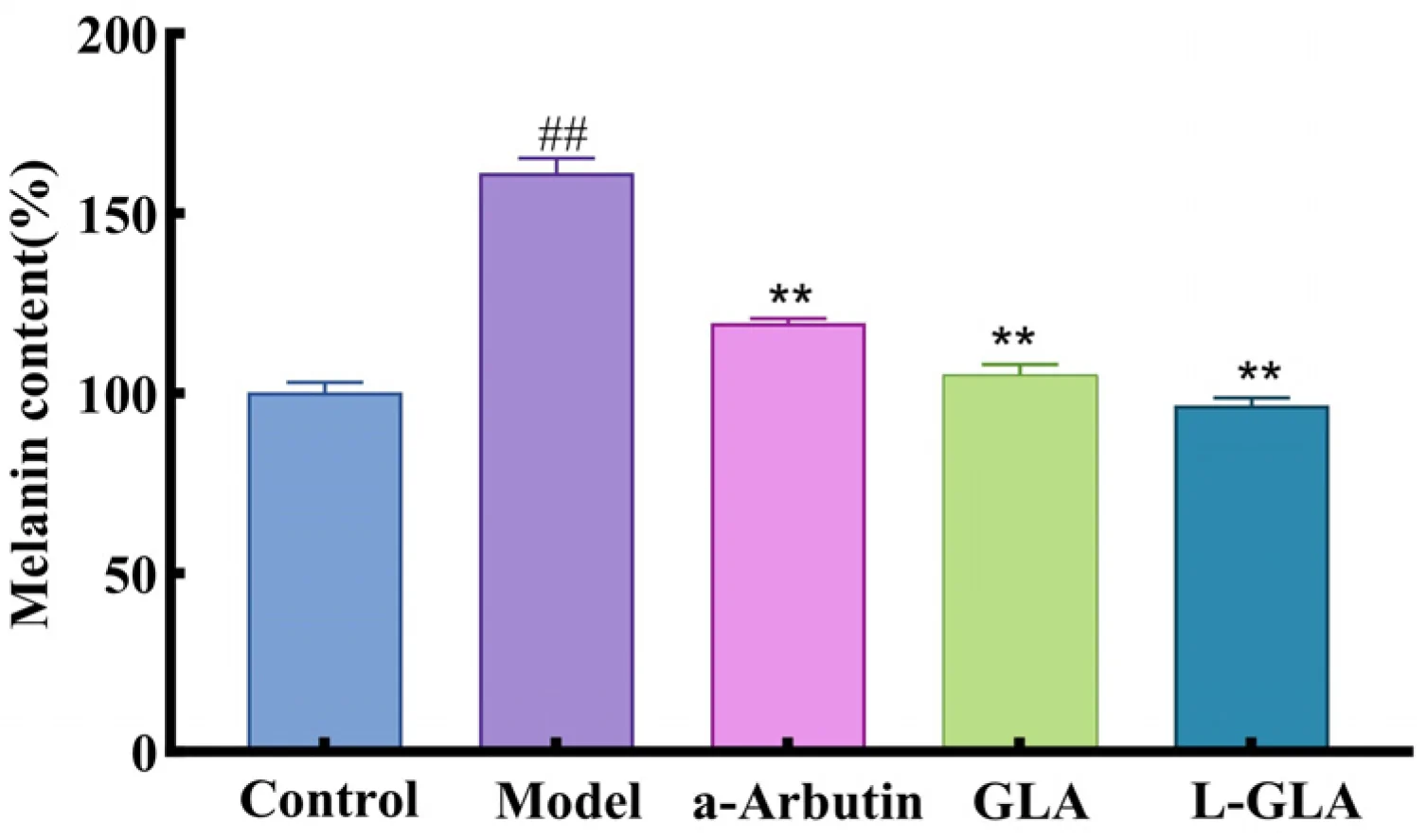
Inhibitory effects of GLA, L-GLA, and α-arbutin on melanin content in α-MSH-stimulated B16 cells. Intracellular melanin content was determined spectrophotometrically and expressed as mean ± SD (n = 3). Annotations: ^##^ *p* < 0.01 vs. control group; ** *p* < 0.01 vs. model group.

To explore the inhibitory effects of GLA and L-GLA on melanogenesis, intracellular melanin content was quantified. As shown in Figure 11, compared with the control group, the melanin content in the model group was higher (*p* < 0.01), suggesting that the melanin-producing cell model was established. Compared with the model group, the melanin content in the α-arbutin (0.5 mmol/L), GLA (32 μM), and L-GLA (32 μM) groups was significantly lower (*p* < 0.01), suggesting that these compounds exert an inhibitory effect on melanin production.

### 2.9. Assay of Cellular TYR Activity

**Figure 12:**
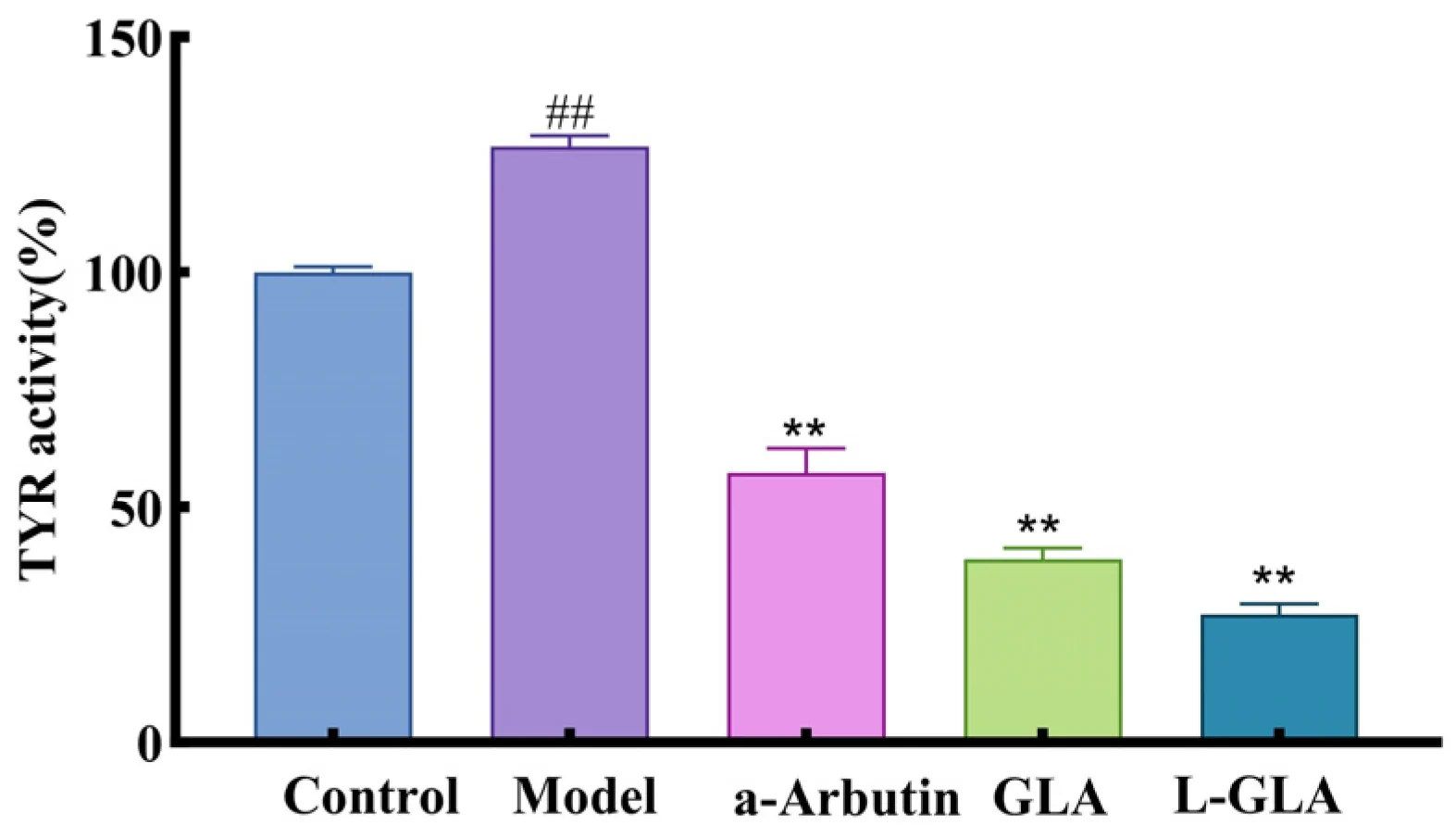
Inhibitory effects of GLA, L-GLA, and α-arbutin on TYR activity in α-MSH-stimulated B16 cells. Data are expressed as mean ± SD (n = 3). Annotations: ^##^ *p* < 0.01 vs. control group; ** *p* < 0.01 vs. model group.

As shown in Figure 12, TYR activity in the model group was higher than that in the control group (*p* < 0.01), suggesting that the melanocyte model was successfully established. In addition, TYR activity in the α-arbutin (0.5 mmol/L), GLA (32 μM), and L-GLA (32 μM) groups was lower than that in the model group (*p* < 0.01), indicating that these compounds inhibit TYR activity.

### 2.10. Real-Time Quantitative PCR

**Figure 13:**
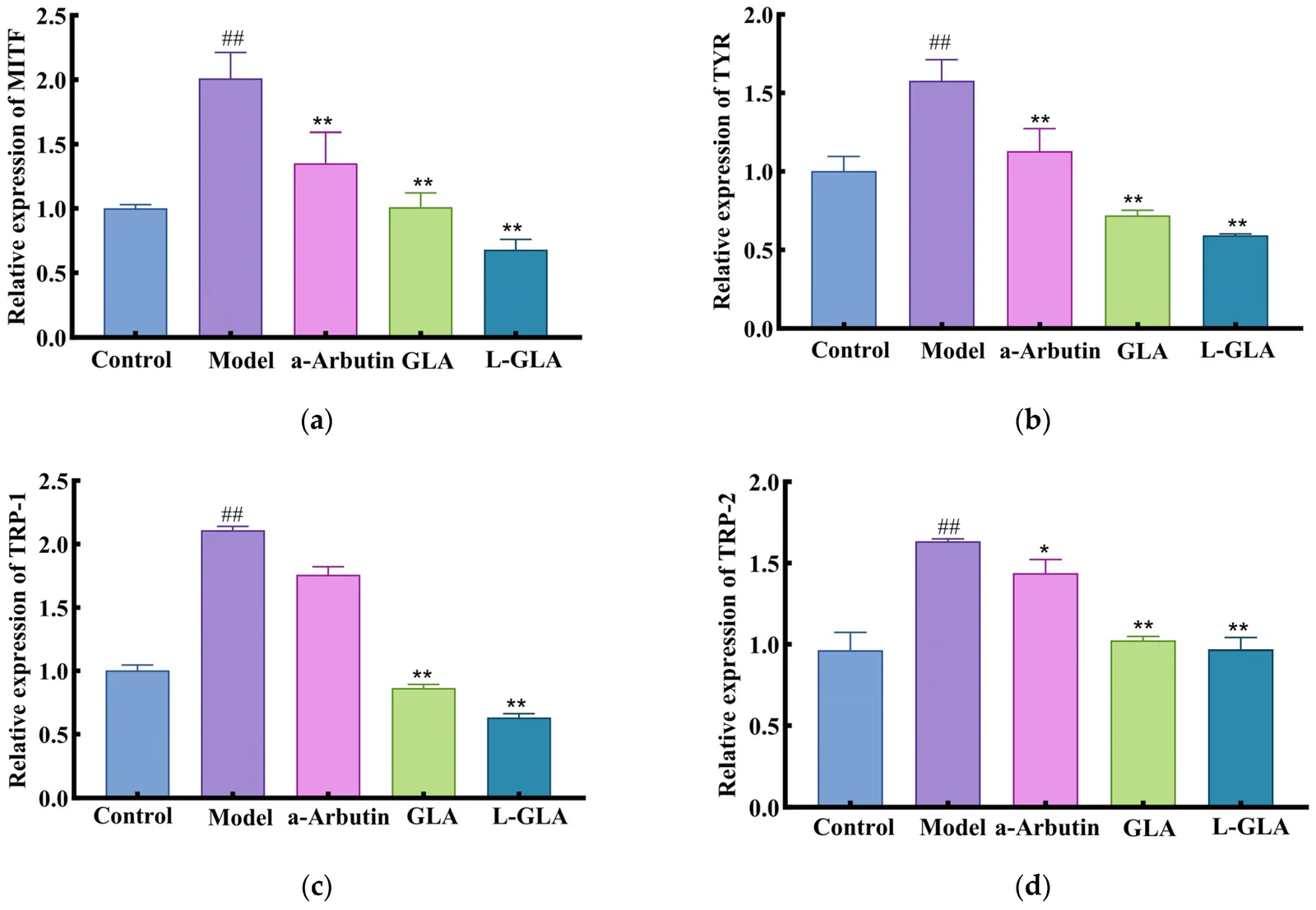
Regulation of MITF (**a**), TYR (**b**), TRP-1 (**c**), and TRP-2 (**d**) gene expression by GLA, L-GLA, and α-arbutin in B16 cells stimulated with α-MSH. Relative mRNA expression levels were analyzed by qRT-PCR. Data are shown as mean ± SD (n = 3). Annotations: ^##^ *p* < 0.01 vs. control group; * *p* < 0.05, ** *p* < 0.01 vs. model group.

As shown in Figure 13, the mRNA expression levels of MITF, TYR, TRP-1, and TRP-2 were higher in the model group than in the control group (*p* < 0.01), indicating an association between these genes and the increased melanin production in the model system. Treatment with α-arbutin (0.5 mmol/L), GLA (32 μM), or L-GLA (32 μM) downregulated the expression of these genes to different extents. GLA and L-GLA reduced all four transcripts (*p* < 0.01), whereas α-arbutin reduced MITF, TYR, and TRP-2 (*p* < 0.01 or *p* < 0.05). Collectively, these findings indicate that GLA and L-GLA inhibit melanogenesis, at least in part, through the downregulation of MITF, TYR, TRP-1, and TRP-2 expression. This regulatory effect may involve the PKA/MITF and MAPK/MITF signaling pathways, though further mechanistic studies are warranted.

### 2.11. Western Blotting

**Figure 14:**
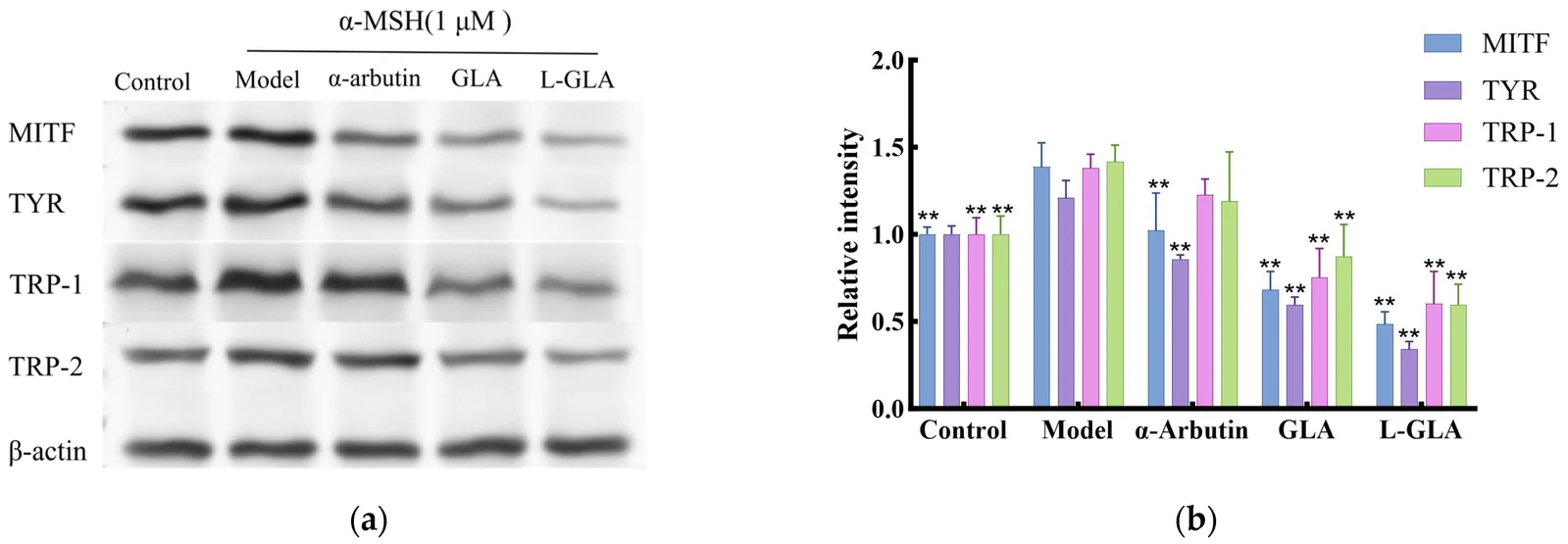
Effects of GLA and L-GLA on α-MSH-induced protein expression of TYR, TRP-1, TRP-2, and MITF in B16 cells. (**a**) Representative Western blot bands of MITF, TYR, TRP-1, TRP-2, and β-actin. (**b**) Quantitative densitometric analysis of protein expression levels normalized to β-actin; data are presented as mean ± SD (n = 3). ** *p* < 0.01 vs. model group.

The protein expression levels of TYR, TRP-1, TRP-2, and MITF were examined by Western blotting. As shown in Figure 14, α-MSH treatment increased the expression of these proteins, whereas co-treatment with GLA or L-GLA counteracted this effect, leading to reduced protein levels of all four factors. Furthermore, GLA and L-GLA exhibited stronger inhibitory activity than α-arbutin under the present experimental conditions. Taken together, these results indicate that GLA and L-GLA suppress melanogenesis through the downregulation of TYR, TRP-1, TRP-2, and MITF, potentially involving the PKA/MITF and/or MAPK/MITF signaling pathways.

## 3. Discussion

L-GLA was prepared using a combination of the film hydration method and high-pressure homogenization. The resulting sub-100 nm particle size and narrow polydispersity (PDI < 0.3) are favorable for enhanced skin penetration and cellular internalization, as liposomes within this size range are known to bypass biological barriers more effectively than larger vesicles [21]. This size reduction is predominantly attributable to the high shear forces generated during homogenization, which effectively disrupt multilamellar vesicles into smaller unilamellar structures. The cumulative release of GLA from L-GLA was higher than that of free GLA, likely because liposomal encapsulation increases the dispersion and effective surface area of GLA in the aqueous medium [10]. Regarding the release behavior, the dissolution of free GLA was well described by the zero-order kinetic model (R^2^ = 0.9785), suggesting a constant-rate process governed by the intrinsic dissolution of its poorly soluble solid particles [40,41]. In contrast, the release of GLA from L-GLA could be adequately described by the Ritger–Peppas model (R^2^ = 0.9556), indicating an anomalous (non-Fickian) transport mechanism [33,42,43]. Beyond release modulation, liposomal encapsulation improved the chemical stability of GLA. This protective effect was further supported by the higher retention of intact GLA in the L-GLA formulation than in free GLA under identical storage conditions (4 °C and 25 °C), which is consistent with the findings of Jovanović et al. [44]. This enhanced stability is likely attributable to the sequestration of the labile 5,8-dihydroxy-1,4-naphthoquinone structure within the hydrophobic core of the phospholipid–cholesterol bilayer, thereby protecting it from thermal oxidative stress [45,46].

Given the improved physicochemical profile of L-GLA, we investigated its biological effects on B16 melanocytes. At concentrations ≤ 32 μM, both agents maintained cell viability above 85%, corroborating their previous cytotoxicity profiles [47]. The differential impact of these compounds on cell cycle distribution suggests distinct mechanisms of anti-proliferative action. While GLA, L-GLA, and α-arbutin each induced G0/G1 phase arrest, L-GLA exhibited the greatest potency, alongside a unique additional reduction in the G2/M population. With respect to the underlying mechanism, the G0/G1 arrest induced by α-arbutin has been reported to be associated with the downregulation of cyclin D1 and upregulation of p21/p27, which may lead to retinoblastoma protein (Rb) hypophosphorylation and subsequent cell cycle blockade [48]. For GLA, our observed G0/G1 blockade appears to be consistent with its known interference with cyclin/CDK complexes in oral cancer models [49]. Of particular note, the unique G2/M perturbation observed exclusively in L-GLA-treated cells may not be solely attributed to the enhanced intracellular bioavailability conferred by the nanoscale size and modified release kinetics, given that the known targets of GLA primarily converge on the G1/S checkpoint. One possibility is that liposomal encapsulation alters the subcellular trafficking of GLA, exposing it to mitotic regulators (e.g., spindle assembly checkpoint proteins or microtubule dynamics). Alternatively, the formulation components (phospholipid/cholesterol) may exert unrecognized synergistic or off-target effects. Therefore, the precise molecular machinery underlying this phase-specific blockade would warrant dedicated investigation in future studies.

In parallel with their effects on cell proliferation, GLA and L-GLA suppressed melanogenesis in α-MSH-stimulated B16 cells. Melanin content and TYR activity were both reduced upon treatment. Critically, all melanin and TYR activity data were rigorously normalized to cell viability during analysis, which largely excludes the possibility that the observed reduction in pigmentation is merely a secondary consequence of decreased cell number. Given the high cell viability (>85%) across all treatment groups, this normalization procedure reinforces that the observed anti-melanogenic effects are independent of cytotoxic artifacts. Taken together, the anti-melanogenic effects may be at least partially related to the transcriptional/translational suppression of TYR protein expression, which is in line with the established role of TYR as the rate-limiting enzyme that catalyzes the initial oxidation of tyrosine to dopaquinone [22,50,51]. To gain insight into the molecular basis of this inhibition, we examined the expression of key melanogenic regulators at both the transcriptional and translational levels. qPCR and Western blot analyses revealed that GLA and L-GLA were associated with downregulated mRNA and protein levels of MITF, TYR, TRP-1, and TRP-2, counteracting the α-MSH-induced upregulation of these factors. Notably, both GLA and L-GLA exerted more potent suppressive effects than α-arbutin at the concentrations tested.

Collectively, our findings suggest that liposomal encapsulation serves as an effective strategy to enhance the physicochemical stability and cellular bioavailability of GLA. The anti-melanogenic activity observed herein likely involves the transcriptional/translational suppression of the MITF axis and a reduction in overall cellular TYR function. Moreover, L-GLA exhibited superior anti-proliferative effects, with a unique impact on the G2/M phase, which supports the pharmacological advantage of the nanoscale formulation. However, this study has inherent limitations: the precise molecular targets of GLA within the cell cycle machinery (e.g., specific CDKs/cyclins) and the upstream signaling pathways modulating MITF remain unidentified. Furthermore, it remains to be elucidated whether the G2/M blockade observed for L-GLA reflects a direct pharmacological effect of GLA on mitotic regulators or an indirect consequence of G0/G1 synchronization, as well as whether the liposomal components contribute to this phenomenon. Despite these limitations, our work provides a preliminary mechanistic framework for the anti-pigmentary effects of GLA and underscores the therapeutic potential of liposome-based delivery of hydrophobic phytochemicals for dermatological applications.

## 4. Materials and Methods

### 4.1. Chemicals and Materials

GLA (purity 93.14%) was prepared in our laboratory. Soybean phospholipids and cholesterol were purchased from Shanghai Taiwei Pharmaceutical Co., Ltd. (Shanghai, China) and Shanghai Lanji Technology Development Co., Ltd. (Shanghai, China), respectively. Acetonitrile and glacial acetic acid (HPLC-grade) were obtained from Sigma-Aldrich (St. Louis, MO, USA). Staurosporine, L-tyrosine (MedChemExpress, Monmouth Junction, NJ, USA), and L-DOPA (Shanghai Yuanye Bio-Technology Co., Ltd., Shanghai, China) were also used. MEM basal medium and fetal bovine serum were supplied by Wuhan Punosai Life Science and Technology Co., Ltd. (Wuhan, China). Purified deionized water was prepared in the laboratory and filtered through a microfiltration membrane before use. Primary antibodies against MITF, TYR, TRP-1, TRP-2, and β-actin were obtained from Abcam (Cambridge, UK), and horseradish peroxidase (HRP)-conjugated anti-rabbit secondary antibody was purchased from Solarbio (Beijing, China). The BCA protein assay kit and RIPA lysis buffer were obtained from Thermo Fisher Scientific (Waltham, MA, USA) and Beyotime (Shanghai, China), respectively. B16 mouse melanoma cells (Wuhan Punosai Life Science and Technology Co., Ltd., Wuhan, China) were cultured in DMEM supplemented with 10% FBS and 1% penicillin–streptomycin at 37 °C in a humidified atmosphere containing 5% CO_2_, and cells from passages 5–10 were used for all experiments. All other reagents were of analytical grade.

### 4.2. Preparation and Characterization of L-GLA

L-GLA samples were prepared using a combination of thin-film hydration and high-pressure homogenization. Briefly, GLA (10 mg), soybean phosphatidylcholine (30 mg), and cholesterol (5 mg) were dissolved in 10 mL of anhydrous ethanol solvent, and the solution was dried under vacuum at 40 °C using a rotary evaporator to remove the organic solvent. After a thin film was formed, it was hydrated with 0.9% sodium chloride solution in a vortex mixer. The liposome was sonicated using a probe sonicator (JY98-IIDN; Scientz, Ningbo, China), and was homogenized three times with a high-pressure homogenizer (KQ5200; Kunshan Ultrasonic Instruments Co., Ltd., Kunshan, China) to decrease particle size and obtain homogeneous vesicles. The solution was filtered through a 0.22 μm polycarbonate filter (Millipore, Billerica, MA, USA) to obtain L-GLA. Blank liposomes (L-BLANK) were prepared using the same method but without GLA. The GLA content in L-GLA was analyzed by reverse-phase high-performance liquid chromatography (HPLC; Shimadzu, Kyoto, Japan). The chromato-graphic system consisted of a C18 column (4.6 × 150 mm, 5 μm). The samples were isolated at a flow rate of 1.0 mL/min, using a mobile phase consisting of acetonitrile/2% acetic acid (56%: 44%, *v*/*v*), with an injection volume of 20 μL, and detected at 282 nm with an ultraviolet–visible detector. The drug loading capacity (LC) was defined as the weight ratio of the loaded drug to the drug-loaded liposomes. The drug encapsulation efficiency (EE) was calculated from the weight ratio of the drug incorporated in the liposomes and the total amount added to the formulation.

The mean diameter and polydispersity index (PDI) of the liposomes were determined by dynamic light scattering. The morphology was assessed using transmission electron microscopy (TEM) without negative staining. Briefly, droplets (15–30 uL) of a liposome suspension were placed on a Formvar-coated copper grid (230 mesh, hexagonal fields). Then, the grids were dried at room temperature, and the morphology of the liposomes was observed by TEM.

### 4.3. The Release Profiles of GLA and L-GLA In Vitro

The release profiles of GLA and L-GLA were determined by a dialysis method. The L-GLA solution (1.0 mL) and the GLA oil solution (1.0 mL) (GLA in soybean oil, concentration equal to that in the liposome) were transferred into a dialysis bag (molecular weight cutoff, 8000–14,000 Da), which was immersed in 10 mL of PBS (pH 7.4) containing 0.5% (*v*/*v*) Tween-80 to maintain a sink condition (37 °C, 100 rpm). At appropriate intervals, 1 mL of the release medium was collected and replaced with 1 mL of a fresh medium. The GLA content in the release medium was then quantified as mentioned above, and the percentage of cumulative release was calculated. All release tests were performed in triplicate. The release kinetic equation of GLA and L-GLA were fitted using zero-order, first-order, Higuchi, and Ritger–Peppas kinetic models. Subsequently, the cumulative release–time curves were plotted.

### 4.4. Establishment of a Temperature Degradation Kinetic Model

GLA and L-GLA were each diluted with ethanol and distilled water, respectively, to identical concentrations and transferred into separate 10 mL centrifuge tubes. The tubes were then incubated at 4 °C, 25 °C, 37 °C, and 45 °C. At 0, 2, 4, 6, 8, 24, and 48 h, samples were withdrawn, diluted to a predetermined dilution factor, and analyzed by high-performance liquid chromatography (HPLC) to determine GLA content. The retention rates of GLA were subsequently calculated from the HPLC data.

The degradation rate constant (K) for GLA is calculated using Equation (1):

(1) $$K=-ln(C/C_{0})/t$$

where C is the GLA concentration (μg/mL) at time t(h), C_0_ is the initial GLA concentration (μg/mL), and k is the degradation rate constant (h^−1^).

The temperature dependence of GLA degradation was evaluated using the Arrhenius equation. By plotting the natural logarithm of the degradation rate constant (lnK) against the reciprocal of the absolute temperature (1/T), the apparent activation energy (Ea) of GLA was calculated from the slope of the resulting curve. The Arrhenius equation can be expressed using Equation (2) as follows:

(2) $$\mathrm{lnK}=-\frac{E_{a}}{\mathrm{RT}}+\ln A$$

where K is the degradation rate constant (h^−1^), Ea is the apparent activation energy (kJ/mol), R is the ideal gas constant (8.314 J/(K·mol)), T is the absolute temperature (K), and A is the Arrhenius constant (pre-exponential factor).

Using Equations (3)–(5), the enthalpy change (ΔH), Gibbs free energy change (ΔG), and entropy change (ΔS) during the GLA degradation process were calculated at different temperatures:

(3) $$\Delta H=E_{a}-\mathrm{RT}$$

(4) $$\Delta G=-\mathrm{RTln}(K_{h}/K_{B})$$

(5) ΔS=(ΔH−ΔG)/T

where Ea is the apparent activation energy (kJ/mol), R is the ideal gas constant (8.314 J/(Kmol)), T is the absolute temperature (K), K is the degradation rate constant (s^−1^), h is Planck’s constant (6.62607015 × 10^−34^ J·s), and kB is Boltzmann’s constant (1.380649 × 10^−23^ J/K).

### 4.5. Cytotoxicity Test

The cytotoxic effects of GLA, L-GLA, L-BLANK (vehicle control), and α-arbutin (positive control) against B16 cells were evaluated using the MTT assay. Briefly, B16 cells were seeded into 96-well plates at a density of 2 × 10^3^ cells per well in 100 μL of medium and incubated at 37 °C in a humidified atmosphere containing 5% CO_2_ for 24 h to allow complete attachment. After adhesion, the cells were treated with 25 μL of serial dilutions of GLA (final concentrations: 144.0, 96.0, 64.0, 32.0, 16.0, 8.0, and 4.0 μmol/L), L-GLA, L-BLANK (final concentrations: 460.37, 383.64, 191.82, 95.91, 47.96, and 23.98 μg/mL), and α-arbutin (final concentrations: 1.8, 1.5, 1.25, 1.0, 0.5, and 0.25 mmol/L), with three replicate wells per concentration. Following 24 h of incubation, 32 μL of MTT solution (5 mg/mL in phosphate-buffered saline) was added to each well, and the plates were further incubated for 4 h at 37. The supernatant was then carefully aspirated, and the formazan crystals were dissolved in 200 μL of dimethyl sulfoxide (DMSO); the plates were shaken on a microplate shaker for 10 min to ensure complete dissolution. The absorbance of each well was recorded at 570 nm using an x-Mark Microplate Absorbance Spectrophotometer (Bio-Rad Life Medical Products (Shanghai) Co., Ltd., Shanghai, China). Cell viability was calculated according to Formula (6):

(6) $$Cell\;viability(\%)=\frac{A-A_{b}}{A_{c}-A_{b}}\times 100\%$$

where A is the absorbance of the sample well, A_b_ is the absorbance of the blank control (medium without cells), and Ac is the absorbance of the untreated control (cells with medium, without any test compound).

The half-maximal inhibitory concentration (IC_50_) values were determined by fitting the concentration–response data to a four-parameter logistic (4PL) nonlinear regression model using GraphPad Prism 9.0 (GraphPad Software, San Diego, CA, USA), according to Equation (7).

(7) $$Y=\mathrm{Bottom}+(\mathrm{Top}\;-\;\mathrm{Bottom})/(1+10\;\times \;\left(\mathrm{Log}{\mathrm{IC}}_{50}\;-\;X\right)\;\times \;\mathrm{Hillslope}))$$

where Y is the percentage of cell viability, X is the log10-transformed compound concentration (µg/mL), Top and Bottom are the upper and lower plateaus of the viability curve, respectively, and Hillslope is the slope factor (Hill coefficient).

### 4.6. Flow Cytometric Analysis of Cell Cycle Distribution and Apoptosis

#### 4.6.1. Cell Cycle Analysis

The B16 cells were seeded at a density of 1 × 10^5^ cells per well in a 6-well plate and cultured for 24 h. The cells were then treated with 32 μM GLA, 32 μM L-GLA, or 0.5 mmol/L α-arbutin and incubated for 48 h. After incubation, the cells were collected into 1.5 mL centrifuge tubes, centrifuged at 300× *g* for 5 min, and the supernatant was discarded. The cells were then washed once with pre-chilled phosphate-buffered saline (PBS), centrifuged again, and 5 × 10^5^ cells were collected. After discarding the supernatant, 1 mL of pre-chilled 70% ethanol was added, and the cells were fixed overnight at 4 °C. Subsequently, the cells were centrifuged at 300× *g* for 5 min, and the supernatant was removed. The cell pellet was resuspended in 1 mL of PBS and allowed to stand at room temperature for 15 min, followed by centrifugation and removal of the supernatant. Next, 100 µL of RNase A reagent was added to fully resuspend the cells, and the suspension was incubated in a 37 °C water bath for 30 min. Then, 400 µL of PI reagent was added, the mixture was thoroughly mixed, and incubation was continued at 2–8 °C in the dark for 30 min. Finally, the cell cycle analysis was performed using a CytoFLEX Flow Cytometer (Beckman Coulter, Inc., Brea, CA, USA) equipped with a 488 nm argon laser. Cells were first gated on a forward scatter (FSC) versus side scatter (SSC) plot to exclude cellular debris, and doublets were excluded by gating on FSC-A versus FSC-H. Propidium iodide (PI) fluorescence was detected through a 585/42 nm bandpass filter (PE channel). For each sample, a total of 1 × 10^4^ events were acquired, and DNA content histograms were analyzed to determine the percentage of cells in G_0_/G_1_, S, and G_2_/M phases. Data analysis was performed using FlowJo software (version 10.8.1, BD Biosciences, San Jose, CA, USA).

#### 4.6.2. Detection of Apoptosis

B16 cells in the logarithmic growth phase were seeded into 6-well plates at a density of 1 × 10^5^ cells per well and cultured for 24 h. The cells were then treated with complete medium containing 32.0 μM GLA, 32.0 μM L-GLA, or 0.5 mmol/L α-arbutin, respectively, and incubated for 48 h. As a positive control for apoptosis induction, cells were treated with 1 μM staurosporine for 6 h. After incubation, the cells were collected into 1.5 mL centrifuge tubes, centrifuged at 300× *g* for 5 min, and the supernatant was discarded. The cells were washed once with pre-chilled phosphate-buffered saline (PBS), centrifuged again, and 5 × 10^5^ cells were collected. After discarding the supernatant, the cell pellet was resuspended in 500 μL of 1× Annexin V binding buffer. Then, 5 μL of Annexin V-FITC and 5 μL of propidium iodide (PI) were added sequentially. The mixture was gently vortexed and incubated at room temperature in the dark for 15 min. Apoptosis was immediately analysed using a CytoFLEX Flow Cytometer (Beckman Coulter, Inc., Brea, CA, USA) equipped with a 488 nm argon laser. FITC fluorescence was detected through a 525/40 nm bandpass filter (FITC channel), and PI fluorescence was detected in the FL3 channel (PE-Cy5-A). Cells were first gated on a forward scatter (FSC) versus side scatter (SSC) plot to exclude cellular debris, and doublets were excluded by gating on FSC-A versus FSC-H. A total of 1 × 10^4^ events were acquired per sample. Fluorescence compensation was performed using single-stained controls (Annexin V-FITC only and PI only). The percentages of viable (Annexin V-FITC^−^/PI^−^), early apoptotic (Annexin V-FITC^+^/PI^−^), late apoptotic (Annexin V-FITC^+^/PI^+^), and necrotic (Annexin V-FITC^−^/PI^+^) cells were determined by quadrant analysis on biparametric dot plots of Comp-FL1-A (FITC-A) versus Comp-FL3-A (PE-Cy5-A). Data analysis was performed using FlowJo software (version 10.8.1, BD Biosciences, San Jose, CA, USA). All experiments were performed in triplicate, and the results are presented as mean ± SD.

### 4.7. Determination of TYR Inhibitory Activity of GLA and L-GLA In Vitro

L-Tyrosine and L-DOPA were employed as substrates for the monophenolase and diphenolase activity assays, respectively. Aliquots (100 μL) of GLA or L-GLA at 10.0, 15.0, 20.0, 25.0, and 30.0 μM, or α-arbutin at 0.5, 1.0, 2.0, 3.0, 4.0 mM, were mixed with 100 μL of mushroom tyrosinase (200 U/mL) in a 96-well plate and preincubated at 25 °C for 10 min. The reaction was subsequently initiated by adding the respective substrate (2.0 mmol/L L-tyrosine for monophenolase or 1.0 mmol/L L-DOPA for diphenolase activity), followed by incubation at 25 °C for 25 min. Absorbance at 475 nm was then recorded using an x-Mark Microplate Absorbance Spectrophotometer (Bio-Rad Life Medical Products (Shanghai) Co., Ltd., Shanghai, China).

### 4.8. Effects on Intracellular Melanin Production

To assess intracellular melanin content, B16 cells were cultured in MEM basal medium supplemented with 10% fetal bovine serum and 1% penicillin–streptomycin at 37 °C in a humidified atmosphere containing 5% CO_2_. Cells at passages 5–10 were used. Logarithmic-phase cells were seeded into 6-well plates at 2 × 10^5^ cells per well and allowed to adhere for 24 h. The medium was then replaced with fresh complete medium containing either 32.0 μM GLA, 32.0 μM L-GLA, 0.5 mmol/L α-arbutin, or 1 μM α-MSH. A blank group (medium alone) and a control group (medium with 1 μM α-MSH) were included, each in triplicate. After 48 h, the supernatant was removed, and the cells were washed twice with ice-cold phosphate-buffered saline (PBS, pH 7.4). Cells were detached with 0.25% trypsin–EDTA, neutralized with complete medium, and gently pipetted to obtain single-cell suspensions. An aliquot was taken for viable cell counting using a hemocytometer under an inverted digital microscope (AE-2000, Motic China Group Co., Ltd., Xiamen, China). The remaining suspension was centrifuged at 1000× *g* for 5 min using a refrigerated centrifuge (2-16R, Hunan Henuo Instrument Equipment Co., Ltd., Changsha, China), and the pellet was lysed with 100 μL of 1 mol/L NaOH solution containing 10% dimethyl sulfoxide, vortexed, and incubated at 80 °C for 30 min. The lysates were transferred to a 96-well plate, and the absorbance at 405 nm was measured with a microplate reader (XMark, Bio-Rad Life Medical Products (Shanghai) Co., Ltd., Shanghai, China). The melanin content was calculated according to Equation (8). All data were normalized to viable cell counts and presented as percentages relative to the control group.

(8) $$Melanin\;content(\%)=\frac{A_{t}-A_{0}}{A_{c}-A_{0}}\;\times \;100\%$$

where A_t_, A_0_, and A_c_ represent the absorbances of the sample, blank, and control groups, respectively. Specifically, the sample group contains cells, culture medium, substrate, and the test sample; the blank group contains only culture medium and substrate (no cells); and the control group contains cells, culture medium, and substrate without the test sample.

### 4.9. Assay of Cellular TYR Activity

B16 cells were seeded at 2 × 10^5^ cells per well in 6-well plates and cultured for 24 h. After washing with PBS, cells were treated with complete medium containing 32.0 μM GLA, 32.0 μM L-GLA, 0.5 mmol/L α-arbutin, or 1 μM α-MSH, with a blank (medium only) and a model control (1 μM α-MSH) included. After 48 h of co-culture, cells were washed, trypsinized, and counted as described in Section 4.7. The pelleted cells were resuspended in lysis buffer (PBS containing 1% Triton X-100, pH 7.4), vortexed, and subjected to one freeze–thaw cycle (−80 °C for 20 min, then 37 °C for 20 min). The lysates were centrifuged at 14,010× *g* for 10 min at 4 °C (2-16R centrifuge), and the supernatants (crude enzyme extracts) were collected. For the reaction, 100 μL of each supernatant was mixed with 100 μL of 5 mmol/L L-DOPA in PBS (pH 6.8) and incubated at 37 °C in the dark for 1 h. Absorbance at 475 nm was recorded using the X Mark microplate reader (Bio-Rad Life Medical Products (Shanghai) Co., Ltd., Shanghai, China). A sample blank was prepared by replacing L-DOPA with PBS. The TYR activity was calculated according to Equation (9). Experiments were performed in three independent replicates, and data are presented as mean ± SD.

(9) $$TYR\;activity(\%)=\frac{A_{t}-A_{0}}{A_{C}-A_{0}}\times 100\%$$

where A_t_, A_0_, and Ac denote the absorbances of the sample, blank, and control groups, respectively. The sample group contains cells, medium, substrate, and the test compound; the blank group contains only medium and substrate (without cells); and the control group contains cells, medium, and substrate but no test compound.

### 4.10. Real-Time Quantitative PCR

#### 4.10.1. RNA Extraction and Reverse Transcription

B16 cells were seeded in 6-well plates at a density of 2 × 10^5^ cells per well and cultured for 24 h. Complete medium containing 32.0 μM GLA, L-GLA, or 0.5 mmol/L α-arbutin was added to the wells, and the cells were incubated for 48 h. The supernatant was discarded, followed by washing three times with PBS; then, 500 μL of Trizol and 100 μL of chloroform were added to each sample, and they were shaken vigorously then left to stand on ice for 2 min. They were then centrifuged at 15,115× *g* for 15 min at 4 °C; the upper aqueous phase was collected, and an equal volume of isopropanol was added. Mixing was performed by shaking and then the mixtures were left to stand at room temperature for 20 min. They were then centrifuged at 15,115× *g* for 10 min at 4 °C, then the supernatant was discarded. The pellet was treated with 1 mL of 75% ethanol to resuspend the pellet at the bottom of the Eppendorf tube. This was centrifuged at 7500× *g* for 5 min at 4 °C, the supernatant was discarded, and it was left to air-dry for 5 min at room temperature. RNase water was added to dissolve the RNA. Reverse transcription was performed using the EasyScript First-Strand cDNA Synthesis SuperMix (TransGen Biotech, Beijing, China) according to the manufacturer’s instructions. Reagents were added according to the reverse transcription kit instructions (Table 5) and mixed by vortexing. PCR amplification was performed according to the reverse transcription protocol (Table 6), thereby converting RNA into cDNA. The obtained cDNA was stored at −20 °C until use.

#### 4.10.2. RT-qPCR

RT-qPCR was performed using a 2× SYBR^®^ Green master mix on a CFX96 real-time PCR system (Bio-Rad, Hercules, CA, USA). RT-qPCR was performed using the 2× SYBR^®^ Green Supermix system. Reagents and cDNA samples were added in sequence according to the reaction protocol (Table 7). The final primer concentration in each reaction was 1 μmol/L. GAPDH was used as the internal reference gene, as its expression remained stable under all experimental conditions. The amplification program was as follows: initial denaturation at 95 °C for 5 min, followed by 40 cycles of denaturation at 95 °C for 10 s and annealing/extension at 60 °C for 30 s (Table 8). Following amplification, a melting curve analysis was performed from 65 °C to 95 °C with 0.5 °C increments every 5 s to verify the specificity of the amplification products. Each reaction was run in triplicate for each sample, and a no-template control (NTC) was included in each run to exclude contamination. The primer sequences for GAPDH, MITF, TYR, TRP-1, and TRP-2 are listed in Table 9.

Relative gene expression levels were calculated using the 2^−ΔΔCt^ method. Briefly, the Ct value of each target gene was first normalized to that of the internal reference gene GAPDH (ΔCt = Cttarget − CtGAPDH), and then the ΔCt of each treatment group was normalized to that of the control group (ΔΔCt = ΔCttreatment − ΔCtcontrol). The fold change in gene expression was calculated as 2^−ΔΔCt^. All data were normalized to the control group and presented as mean ± SD from three independent experiments.

### 4.11. Western Blotting

B16 cells in the logarithmic growth phase were seeded into 6-well plates at a density of 2 × 10^5^ cells per well and cultured for 24 h, followed by treatment with 32.0 μM GLA, L-GLA, or 0.5 mmol/L α-arbutin for 48 h at 37 °C in a humidified 5% CO_2_ incubator, with six replicate wells per group. After treatment, the culture medium was aspirated, and the cells were washed three times with ice-cold phosphate-buffered saline (PBS); total cellular proteins were subsequently extracted using ice-cold RIPA lysis buffer supplemented with 1 mmol/L phenylmethylsulfonyl fluoride (PMSF), incubated on ice for 30 min, and centrifuged at 12,000× *g* for 10 min at 4 °C. The resulting supernatants were collected, protein concentrations were determined using a BCA protein assay kit, and all samples were normalized to equal protein concentrations, mixed with 5× SDS-PAGE loading buffer, denatured at 65 °C for 15 min, and stored at −80 °C until further analysis. Equal amounts of denatured protein were separated by SDS-PAGE and transferred onto polyvinylidene difluoride (PVDF) membranes (Millipore, Burlington, MA, USA) via wet transfer; the membranes were blocked with 5% bovine serum albumin (BSA) in Tris-buffered saline containing 0.1% Tween-20 (TBST) for 1 h at room temperature and subsequently incubated overnight at 4 °C with primary antibodies against MITF (1:1000), TYR (1:1000), TRP-1 (1:1000), TRP-2 (1:1000), and β-actin (1:1000) as the internal loading control. After three washes with TBST, the membranes were incubated with horseradish peroxidase (HRP)-conjugated anti-rabbit secondary antibody (1:5000) for 90 min at room temperature, followed by five washes with TBST (6 min each); immunoreactive bands were then visualized using an enhanced chemiluminescence (ECL) detection kit (Millipore, USA) and captured using a chemiluminescence imaging system. Band intensities were quantified with ImageJ software (version 1.54f, National Institutes of Health, Bethesda, MD, USA), and relative protein expression levels were normalized to β-actin, with the control group arbitrarily set to 1.0. All experiments were performed in triplicate, and data are presented as the mean ± standard deviation (SD).

### 4.12. Statistical Analysis

All data are presented as mean ± standard deviation (SD), with a minimum of three independent replications per experiment (n ≥ 3). The normality of the data was assessed using the Shapiro–Wilk test, and homogeneity of variances was evaluated by Bartlett’s test. For comparisons between two groups, two-tailed Student’s *t*-test was employed. For multiple-group comparisons, one-way analysis of variance (ANOVA) was performed, followed by Tukey’s post hoc test for equal variances. Statistical significance was set at *p* < 0.05 and indicated as follows: * *p* < 0.05, ** *p* < 0.01. All analyses were conducted using GraphPad Prism 9.0 and Origin software 2022.

## 5. Conclusions

In this study, L-GLA was developed to overcome the inherent limitations of GLA, including its poor aqueous solubility, susceptibility to oxidative degradation, and low bioavailability. L-GLA was prepared via a thin-film hydration method combined with high-pressure homogenization. Physicochemical characterization revealed that the liposomes had a mean particle size of 50.24 ± 1.15 nm, a PDI of 0.297 ± 0.01, and a Zeta potential of −3.44 ± 0.04 mV. The drug loading content and encapsulation efficiency were determined to be 4.38% and 80.71%, respectively. The cumulative release of GLA from L-GLA over 96 h was higher than that of free GLA. Release kinetics analysis showed that the release profile of free GLA followed a zero-order kinetic model (R^2^ = 0.9785), whereas the release behavior of L-GLA was best described by the Ritger–Peppas model (R^2^ = 0.9556). Temperature-dependent degradation kinetic fitting revealed that L-GLA exhibited a lower degradation rate constant (k), along with higher activation energy (E_a_) and activation enthalpy (Δ*H*), compared with free GLA. Taken together, these results indicate that the liposomal formulation possesses superior stability under the conditions tested. In cellular assays, the IC_50_ of L-GLA against B16 melanoma cells (84.66 μM) was lower than that of GLA (115.5 μM), and both compounds induced apoptosis. The potential mechanism may involve inhibition of B16 cell proliferation via G0/G1 phase arrest, suppression of melanin production through inhibition of TYR activity, and transcriptional downregulation of melanogenesis-related genes, including MITF, TRP-1, TRP-2, and TYR. These effects may be associated with the PKA/MITF and MAPK/MITF signaling pathways.

## Figures and Tables

**Table 1:** Release kinetic parameters of GLA with zero-order, first-order, Higuchi, and Ritger–Peppas models.

Fitted Model	Rate Equation	R^2^
Zero-order kinetics	y = 7.96 + 0.50x	0.9785
First-order kinetics	y = 45.62 × (1 − e^−0.04^)	0.8996
Higuchi	y = 5.16x^1/2^ − 1.69	0.9561
Ritger–Peppas	y = 3.96x^0.56^	0.9651

**Table 2:** Release kinetic parameters of L-GLA with zero-order, first-order, Higuchi, and Ritger–Peppas models.

Fitted Model	Rate Equation	R^2^
Zero-order kinetics	y = 7.78 + 0.83x	0.9492
First-order kinetics	y = 73.05 × (1 − e^−0.03^)	0.9277
Higuchi	y = 7.82x^1/2^ − 3.24	0.9390
Ritger–Peppas	y = 4.83x^0.61^	0.9556

**Table 3:** Rate constants and activation energies of GLA and L-GLA at different temperatures.

Temperature/°C	k/h^−1^		R^2^		Ea/(kJ/mol)	
	GLA	L-GLA	GLA	L-GLA	GLA	L-GLA
4	0.008	0.003	0.9061	0.9006	12.63	14.02
25	0.013	0.004	0.9112	0.9027		
37	0.015	0.005	0.9168	0.9211		
45	0.016	0.007	0.9111	0.9045		

**Table 4:** Thermodynamic parameters (ΔH and ΔG) of GLA and L-GLA at different temperatures.

Temperature/°C	ΔH(kJ/mol)		ΔG(kJ/mol)	
	GLA	L-GLA	GLA	L-GLA
4	10.33	11.72	88.22	90.48
25	10.15	11.54	93.89	96.81
37	10.05	11.44	97.40	100.23
45	9.99	11.38	99.81	101.99

**Table 5:** Reverse transcription incubation system.

Reagents	Usage
Total RNA	4 μg
Anchored Oligo (dT) 18 Primer (0.5 μg/μL)	1 μL
2 × ES Reaction Mix	10 μL
Easy Script RT/RI Enzyme	1 μL
gDNA Remover	1 μL
RNase-free water	Variable
Total volume	20 μL

**Table 6:** Reverse transcription program.

Temperature (°C)	Time
42	15 min
85	5 s
12	∞

**Table 7:** Real-time fluorescence quantitative PCR reaction system.

Group	Volume (μL)	Volume (μL)
Hifair^®^ qPCR SYBR Green Master Mix	5	5
Forward Primer	1	1
Reverse Primer	1	1
cDNA	1	1
RNase-free Water	2	2
Total	10	10

**Table 8:** Real time-PCR procedure.

Cyclic Step	Temperature (°C)	Time (s)	Cycle Number
Initial denaturation	95	300	1
Denaturation	95	10	40
Primer annealing	60	30	

**Table 9:** Primer Sequence 1 for RT-PCR.

Genes	Forward Primer (5′ to 3′)	Reverse Primer (5′ to 3′)
GAPDH	TGTGTCCGTCGTGGATCTGA	CCTGCTTCACCACCTTCTTGA
MITF	CAAATGGCAAATACGTTACCCG	CAATGCTCTTGCTTCAGACTCT
TYR	AGCCCAGCATCCTTCTTCTCCTC	AGTGGTCCCTCAGGTGTTCCATC
TRP-1	ATGAAATCTTACAACGTCCTCCC	GCACACTCTCGTGGAAACTGA
TRP-2	GGGGCTTTGATGTACCCTAGC	TGGAGTGGTTAGGATTCGGG

## Data Availability

The original contributions presented in this study are included in the article. Further inquiries can be directed to the corresponding author.

## References

[1] Chung C.-L., Chen J.-H., Huang W.-C., Sheu J.-R., Hsia C.-W., Jayakumar T., Hsia C.-H., Chiou K.-R., Hou S.-M. (2022). Glabridin, a Bioactive Flavonoid from Licorice, Effectively Inhibits Platelet Activation in Humans and Mice. Int. J. Mol. Sci..

[2] Razzak M.A., Lee J.E., Choi S.S. (2019). Structural insights into the binding behavior of isoflavonoid glabridin with human serum albumin. Food Hydrocoll..

[3] Qin H., Wang S., Peng M., Zu Y., Zhang M., Liu K. (2026). German Chamomile-Derived Extracellular Vesicles for the Transdermal Delivery of Glabridin: A Combined Strategy against UVA-Induced Pigmentation. ACS Appl. Nano Mater..

[4] Zhang J., Wu X., Zhong B., Liao Q., Wang X., Xie Y., He X. (2023). Review on the Diverse Biological Effects of Glabridin. Drug Des. Dev. Ther..

[5] Shin J., Choi L.S., Jeon H.J., Lee H.M., Kim S.H., Kim K.-W., Ko W., Oh H., Park H.S. (2023). Synthetic Glabridin Derivatives Inhibit LPS-Induced Inflammation via MAPKs and NF-κB Pathways in RAW264.7 Macrophages. Molecules.

[6] Luo M., Oomah B.D., Akoetey W., Zhang Y., Daneshfozoun H., Hosseinian F. (2024). Liposomes as sustainable delivery systems in food, cosmetic, and pharmaceutical applications. J. Am. Oil Chem. Soc..

[7] Qi J., Wang Y., Chen H., Wu K., Zhou P., Dou Y., Xiong B., Zhou W. (2025). Advancing the Identification of Bioactive Molecules and the Construction of a Synergistic Drug Delivery System in Combating Lung Injury. Adv. Sci..

[8] Kabdraisova A.Z., Umbetova A.K., Kairalapova G.Z., Litvinenko Y.A., Sassykova L.R., Yelibayeva N.S., Burasheva G.S., Berganayeva A.E., Assylkhanov Z.S., Dauletova M.D. (2026). Machine-Learning-Driven Molecular Design and Structure–Property–Performance Relationships in Pharmaceutical Chemistry. Molecules.

[9] Liu F.-C., Yang Y.-H., Liao C.-C., Lee H.-C. (2024). Xanthoxylin Attenuates Lipopolysaccharide-Induced Lung Injury through Modulation of Akt/HIF-1α/NF-κB and Nrf2 Pathways. Int. J. Mol. Sci..

[10] Cheng L., Xu J., Wang A., Hou S., Li A. (2025). Glabridin-encapsulated liposomes targeting melanocytes through the melanocortin 1 receptor. Colloids Surf. B Biointerfaces.

[11] Li C., Wang Y., Zhang W., Yang X., Wang Y., Hou G., Wang D., Han B., Zhang Y. (2024). The antitumor mechanisms of glabridin and drug delivery strategies for enhancing its bioavailability. Front. Oncol..

[12] Burnouf P.-A., Su Y.-C., Yang S.-H. (2025). Safe and Efficient Intracellular Release of SN38 via Lysosomal-Responsive SN38-G Loaded Liposomes. ACS Appl. Bio Mater..

[13] Jash A., Ubeyitogullari A., Rizvi S.S.H. (2021). Liposomes for oral delivery of protein and peptide-based therapeutics: Challenges, formulation strategies, and advances. J. Mater. Chem. B.

[14] Katona G., Sabir F., Sipos B., Naveed M., Schelz Z., Zupkó I., Csóka I. (2022). Development of Lomustine and *n*-Propyl Gallate Co-Encapsulated Liposomes for Targeting Glioblastoma Multiforme via Intranasal Administration. Pharmaceutics.

[15] Jiang Y., Li W., Wang Z., Lu J. (2024). Lipid-Based Nanotechnology: Liposome. Pharmaceutics.

[16] Burdușel A.-C., Andronescu E. (2022). Lipid Nanoparticles and Liposomes for Bone Diseases Treatment. Biomedicines.

[17] Zhou X., Liu E., Li J., Chen F., Zhang Y., Chen K., Qi D. (2023). Facile Synthesis of Rodlike TiO_2_/Lignin Nanostructures as Multifunctional Emulsifiers for High Internal Phase Emulsions. ACS Appl. Nano Mater..

[18] Hu Z., Dong S., Huang J., Zhang L., Hu Z., Wang H., Qin L., Tangthianchaichana J., Lu Y. (2026). From Solubilization to Structural Perturbation: The Mechanism of Polyquaternium-51-Driven Enhanced Transdermal Delivery of Glabridin. Langmuir.

[19] Lu Y., Cheng L., Ren L., Chen D., Guan S., Zhu S., Xu X., Zhang B., Tang M., Zhang C. (2024). Therapeutic Alleviation and Mechanism of Glabridin Liposome on Histamine-induced Atopic Dermatitis. Pharmacogn. Mag..

[20] Kim S.-H., Lee Y.-C. (2022). Plant-Derived Nanoscale-Encapsulated Antioxidants for Oral and Topical Uses: A Brief Review. Int. J. Mol. Sci..

[21] Crowley L.C., Marfell B.J., Scott A.P., Waterhouse N.J. (2016). Quantitation of Apoptosis and Necrosis by Annexin V Binding, Propidium Iodide Uptake, and Flow Cytometry. Cold Spring Harb. Protoc..

[22] Snyman M., Walsdorf R.E., Wix S.N., Gill J.G. (2024). The metabolism of melanin synthesis—From melanocytes to melanoma. Pigment. Cell Melanoma Res..

[23] Zhang J., Wang C., Wang C., Sun B., Qi C. (2018). Understanding the role of extracts from sea buckthorn seed residues in anti-melanogenesis properties on B16F10 melanoma cells. Food Funct..

[24] Logesh R., Prasad S.R., Chipurupalli S., Robinson N., Mohankumar S.K. (2023). Natural tyrosinase enzyme inhibitors: A path from melanin to melanoma and its reported pharmacological activities. Biochim. Biophys. Acta (BBA)—Rev. Cancer.

[25] Lee M.-K., Moon K.M., Park S.-Y., Seo J., Kim A.-R., Lee B. (2024). 10(E)-Pentadecenoic Acid Inhibits Melanogenesis Partly Through Suppressing the Intracellular MITF/Tyrosinase Axis. Antioxidants.

[26] Yang S., Liu B., Ji K., Fan R., Dong C. (2018). MicroRNA-5110 regulates pigmentation by cotargeting melanophilin and WNT family member 1. FASEB J..

[27] Chen J., Fang Q., Liu S., Yang G., Gao Y. (2017). Influences of several factors on the photolysis of glabridin under UV irradiation. J. Photochem. Photobiol. A Chem..

[28] Ding X., Mei E., Hu M., Zhou C., Li X., Cai L., Li Z. (2019). Effect of puerarin on melanogenesis in human melanocytes and vitiligo mouse models and the underlying mechanism. Phytother. Res..

[29] Zhang Y., Hu Y., Lei L., Jiang L., Fu C., Chen M., Wu S., Duan X., Chen J., Zeng Q. (2024). UVB-induced TRPS1 regulates MITF transcription activity to promote skin pigmentation. Biochim. Biophys. Acta (BBA)—Rev. Cancer.

[30] Song W., Zhao Y.-Y., Ren Y.-J., Liu L.-L., Wei S.-D., Yang H.-B. (2021). Proanthocyanidins isolated from the leaves of *Photinia* × *fraseri* block the cell cycle and induce apoptosis by inhibiting tyrosinase activity in melanoma cells. Food Funct..

[31] Shu P., Wang Y., Zhang L. (2024). The Effect of α-Arbutin on UVB-Induced Damage and Its Underlying Mechanism. Molecules.

[32] Laracuente M.-L., Yu M.H., McHugh K.J. (2020). Zero-order drug delivery: State of the art and future prospects. J. Control. Release.

[33] Li W., Chountoulesi M., Antoniadi L., Angelis A., Lei J., Halabalaki M., Demetzos C., Mitakou S., Skaltsounis L.A., Wang C. (2022). Development and physicochemical characterization of nanoliposomes with incorporated oleocanthal, oleacein, oleuropein and hydroxytyrosol. Food Chem..

[34] Biagini A., Refrigeri N., Caglioti C., Sabbatini P., Ticconi S., Ceccarelli G., Iannitti R.G., Palazzetti F., Fioretti B. (2024). Accelerated Stability Testing in Food Supplements Underestimates Shelf Life Prediction of Resveratrol with Super-Arrhenius Behavior. Symmetry.

[35] Yu B., Zeng L., Yang H. (2019). Multivariate Mixed-Effects Kinetic Models for Multiple Correlated Quality Attributes from Accelerated Stability Studies. Stat. Biopharm. Res..

[36] Sheguang D. (2025). Alternative approach to calculate thermodynamic enthalpy change ΔH for non-ideal fluids via equations of state. J. Mol. Liq..

[37] da Silva J.D.F., Breda F.C., Ribeiro L.C., de Lima J.A., Berretta A., Prestes O.D., Zanella R., Cichoski A.J., Mello R.D.O. (2025). Kinetics-thermodynamics of the antioxidant activity of propolis extracts in pork pâté. Food Res. Int..

[38] Li Z., Luo M., Ni L., Zhou Q., Jiang C., Zhu H., Wang Z., Liu Q., Shu P. (2025). The molecular mechanisms underlying Glabridin enhancing the topical permeability and anti-melanogenic effect of α-arbutin. J. Drug Deliv. Sci. Technol..

[39] McKenney C.D., Regot S. (2025). Cell cycle regulation by the ribotoxic stress response. Trends Cell Biol..

[40] Zhang R., Shi H., Li S., Zhang H., Zhang D., Wu A., Zhang C., Li C., Fu X., Chen S. (2023). A double-layered gastric floating tablet for zero-order controlled release of dihydromyricetin: Design, development, and in vitro/in vivo evaluation. Int. J. Pharm..

[41] Adebo O.A., Oyedeji A.B., Adebiyi J.A., Chinma C.E., Oyeyinka S.A., Olatunde O.O., Green E., Njobeh P.B., Kondiah K. (2021). Kinetics of Phenolic Compounds Modification during Maize Flour Fermentation. Molecules.

[42] Cuomo F., Ceglie A., Piludu M., Miguel M.G., Lindman B., Lopez F. (2014). Loading and protection of hydrophilic molecules into liposome-templated polyelectrolyte nanocapsules. Langmuir ACS J. Surf. Colloids.

[43] Fancher I.S., Rubinstein I., Levitan I. (2019). Potential Strategies to Reduce Blood Pressure in Treatment-Resistant Hypertension Using Food and Drug Administration-Approved Nanodrug Delivery Platforms. Hypertension.

[44] Jovanović A.A., Balanč B., Petrović P.M., Čutović N., Marković S.B., Djordjević V.B., Bugarski B.M. (2025). Liposome-Based Encapsulation of Extract from Wild Thyme (*Thymus serpyllum* L.) Tea Processing Residues for Delivery of Polyphenols. Foods.

[45] Rao Y., Li R., Liu S., Meng L., Wu Q., Yuan Q., Liang H., Qin M. (2022). Enhanced bioavailability and biosafety of cannabidiol nanomicelles for effective anti-inflammatory therapy. Particuology.

[46] Nsairat H., Ibrahim A., Jaber A., Abdelghany S., Atwan R., Shalan N., Abdelnabi H., Odeh F., El-Tanani M., Alshaer W. (2023). Liposome bilayer stability: Emphasis on cholesterol and its alternatives. J. Liposome Res..

[47] Pan C., Liu X., Zheng Y., Zhang Z., Li Y., Che B., Liu G., Zhang L., Dong C., Aisa H.A. (2022). The mechanisms of melanogenesis inhibition by glabridin: Molecular docking, PKA/MITF and MAPK/MITF pathways. Food Sci. Hum. Wellness.

[48] Nahar L., Al-Groshi A., Kumar A., Sarker S.D. (2022). Arbutin: Occurrence in Plants, and Its Potential as an Anticancer Agent. Molecules.

[49] Chen C.-T., Chen Y.-T., Hsieh Y.-H., Weng C., Yeh J., Yang S., Lin C., Yang J. (2018). Glabridin induces apoptosis and cell cycle arrest in oral cancer cells through the JNK1/2 signaling pathway. Environ. Toxicol..

[50] Wagatsuma T., Suzuki E., Shiotsu M., Sogo A., Nishito Y., Ando H., Hashimoto H., Petris M.J., Kinoshita M., Kambe T. (2023). Pigmentation and TYRP1 expression are mediated by zinc through the early secretory pathway-resident ZNT proteins. Commun. Biol..

[51] Merecz-Sadowska A., Sitarek P., Kowalczyk T., Zajdel K., Kucharska E., Zajdel R. (2022). The Modulation of Melanogenesis in B16 Cells Upon Treatment with Plant Extracts and Isolated Plant Compounds. Molecules.

